# microRNA-dependent gene regulatory networks in maize leaf senescence

**DOI:** 10.1186/s12870-016-0755-y

**Published:** 2016-03-22

**Authors:** Xiangyuan Wu, Dong Ding, Chaonan Shi, Yadong Xue, Zhanhui Zhang, Guiliang Tang, Jihua Tang

**Affiliations:** National Key Laboratory of Wheat and Maize Crop Science, Collaborative Innovation Center of Henan Grain Crops, College of Agronomy, Henan Agricultural University, 95 Wenhua Road, Zhengzhou, 450002 China; Hubei Collaborative Innovation Center for the Grain Industry, Yangtze University, Jingzhou, 434025 China; Department of Biological Sciences, Michigan Technological University, Houghton, MI 49931 USA

**Keywords:** Maize, Leaf senescence, Deep sequencing, microRNAs, Regulatory mechanism

## Abstract

**Background:**

Maize grain yield depends mainly on the photosynthetic efficiency of functional leaves, which is controlled by an array of gene networks and other factors, including environmental conditions. MicroRNAs (miRNAs) are small RNA molecules that play important roles in plant developmental regulation. A few senescence-associated miRNAs (SA-miRNAs) have been identified as important participants in regulating leaf senescence by modulating the expression levels of their target genes.

**Results:**

To elucidate miRNA roles in leaf senescence and their underlying molecular mechanisms in maize, a stay-green line, Yu87-1, and an early leaf senescence line, Early leaf senescence-1 (ELS-1), were selected as experimental materials for the differential expression of candidate miRNAs. Four small RNA libraries were constructed from ear leaves at 20 and 30 days after pollination and sequenced by Illumina deep sequencing technology. Altogether, 81 miRNAs were detected in both lines. Of these, 16 miRNAs of nine families were differentially expressed between ELS-1 andYu87-1. The phenotypic and chlorophyll content analyses of both lines identified these 16 differentially expressed miRNAs as candidate SA-miRNAs.

**Conclusions:**

In this study, 16 candidate SA-miRNAs of ELS-1 were identified through small RNA deep sequencing technology. Degradome sequencing results indicated that these candidate SA-miRNAs may regulate leaf senescence through their target genes, mainly transcription factors, and potentially control chlorophyll degradation pathways. The results highlight the regulatory roles of miRNAs during leaf senescence in maize.

**Electronic supplementary material:**

The online version of this article (doi:10.1186/s12870-016-0755-y) contains supplementary material, which is available to authorized users.

## Background

Leaf senescence is a major physiological process that affects vegetative and productive developmental processes in plants, and delayed senescence can prolong the leaf life span and increase seed yield [1]. In the leaf senescence process, leaves lose their chloroplasts, convert the photosynthetic products into carbohydrates, and transport them into sink organs, such as seeds [2, 3]. The conversion from leaf maturation to senescence is complex and associated with changes in genome-wide gene expression levels and several senescence-associated genes (SAGs) have been discovered in many plant species [4]. Approximately 5,356 SAGs were identification from 44 species and ~69.89 % were found in *Arabidopsis thaliana*. Additionally, over 100 transcription factors, such as **N**AM, **A**TAF, and **C**UC (NAC), as well as WRKY, SQUAMOSA promoter-binding protein (SBP), APETALA2 (AP2), and MYB, are involved in leaf senescence regulation [5–7].

The phytohormone response pathway is important in the leaf senescence regulatory network. Cytokinin and auxin (AUX) can delay leaf senescence, while ethylene, abscisic acid (ABA), jasmonic acid (JA), and salicylic acid (SA) can promote leaf senescence [8]. Recently, molecular mechanisms involved in the control of leaf senescence by phytohormones have attracted attention. NAC members are transcription factors involved in plant leaf senescence [9]. *NAC2* was induced by *ETHYLENE INSENSITIVE3* (*EIN3*), which could improve leaf senescence in Arabidopsis. AUX is an negative regulatory factor in leaf senescence, while Auxin Response Factor transcription factors could bind to the promoters of the *AUX* genes and regulate their expression levels [10]. Additionally, ABA, JA, SA, and brassinosteroids are involved in regulating plant development and senescence [11–14].

Plant miRNAs are small non-coding RNAs, 21-nucleotide (nt) in length, which play critical roles in the regulation of their target genes by cleaving target transcripts at the post-transcriptional level [15, 16]. MiR164 participates in the leaf senescence feed-forward pathway, together with *EIN3* and *ORESARA1* (*ORE1*) [17]. Recently, chlorophyll catabolic genes (*CCG*s) and *ACS2* (a major ethylene biosynthesis gene) were found to be involved in this feed-forward loop [18]. Both *EIN3* and *ORE1* could directly bind to the promoters of the *CCG*s and activate gene expression during ethylene-mediated chlorophyll degradation. MiR319 is a senescence-associated miRNA that represses the onset of senescence. Five TEOSINTE BRANCHED/CYCLOIDEA/PCF (TCPs) transcription factors were regulated by miR319 and function in leaf developmental regulation [19–21]. Meanwhile, *TCPs* directly regulate lipoxygenase 2 (*LOX2*), which encodes a key enzyme of JA biosynthesis, and a high level of miR319 leads to delayed leaf senescence in Arabidopsis [22]. Additionally, other miRNAs and their targets, for example, osa-miR159, osa-miR160/miR167, osa-miR164,and osa-miR172, targeting mRNAs coding for MYB/TCP, Auxin Response Factor, salicylic acid-induced protein 19, and AP2 proteins, respectively, were also found to be involved in leaf senescence through phytohormone signaling pathways in rice [23].

**Fig. 1:**
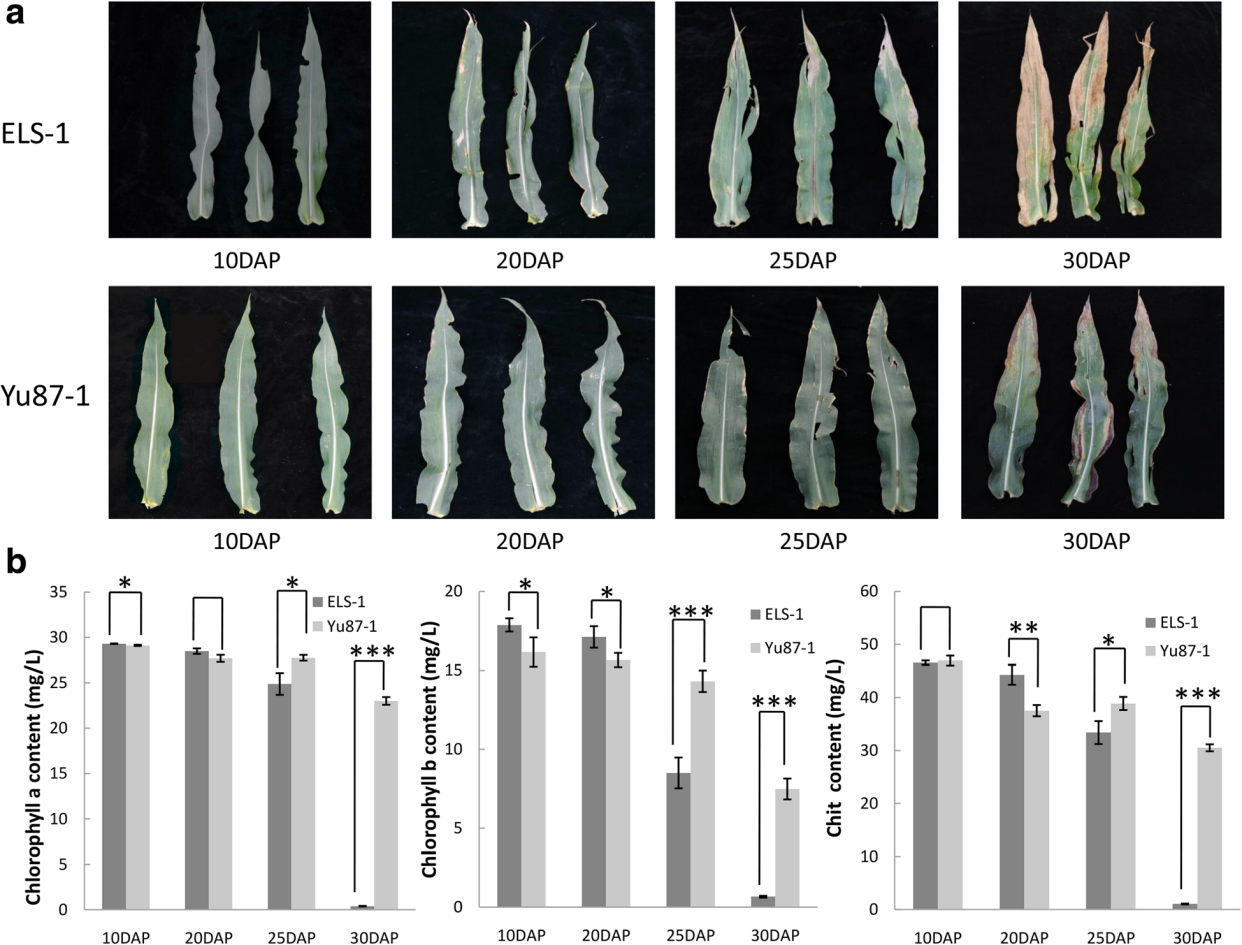
Leaves and chlorophyll content over the course of leaf senescence developmet. **a** Phenotypes of maize ear leaves at different days after pollination (DAP). Each sample was selected randomly from uniform plants. Representative plants were photographed to show the progressive yellowing process of leaves over the lifespan. **b** Chlorophyll content in the leaves of inbred lines ELS-1 and 87-1. The chlorophyll contents decreased with age in both materials but at different rates. The data were derived from triplicate experiments with the standard deviation plotted. CHIT, total chlorophyll content. Bars represent mean ± SE, *n* ≥ 5.^*^, *p* < 0.1;^**^, *p* < 0.01;^***^, *p* < 0.001

Hybrid maize has a long active photosynthetic period that is mainly achieved by having a higher chlorophyll content during senescence, or by maintaining a higher photosynthetic activity level during chlorophyll loss, which increases grain yield. In this study, two inbred lines with distinct leaf senescence characteristics, early leaf senescence, in the namesake Early leaf senescence-1 (ELS-1; Fig. 1), and stay-green, delay-in-leaf-senescence, in the elite inbred line Yu87-1, were selected as the materials to determine the potential roles of miRNAs and their target genes in leaf senescence, and to explore the network between them. The information will increase our understanding of the molecular mechanisms of leaf senescence.

## Results

### Characterization of leaf senescence in two inbred lines

To characterize the senescence behavior of ELS-1 and Yu87-1, the phenotypes of ear leaves at 10DAP, 20DAP, 25DAP, and 30DAP were observed in the field. The leaves of the two lines all remained green and had the same performance from 0 to 10 DAP. However, the leaves of inbred line ESL-1 lost their green color during the 20–25 DAP period, while the leaves of inbred line Yu87-1 stayed green during this stage (Fig. 1). After 30 DAP, the inbred line ESL-1 had already lost most of its leaf chlorophyll, while the top sections inbred line Yu87-1 leaves had just turned yellow.

Interestingly, in the inbred line, ELS-1 yellowing occurred faster on the top part of the leaves than in the middle and base parts from 25 to 30DAP. The chlorophyll *a* content sharply dropped from 25.58mg/L at 25DAP to 0.35 mg/L at 30DAP in the leaves of the inbred line ELS-1, while only a slight reduction of chlorophyll *a*, from 27.41 mg/L to 23.01 mg/L, was observed in the leaves of inbred line Yu87-1 during the same time period. The change in the chlorophyll *b* content was different from that of chlorophyll *a*. In the leaves of inbred line ELS-1, chlorophyll *b* decreased faster and earlier than chlorophyll *a* (Fig. 1). A reduction, from 13.45 mg/L to 2.30 mg/L, of chlorophyll *b* was detected at 20DAP in ELS-1. The total chlorophyll content decreased in both inbred lines with age, and the variation trend was more acute in the leaves of inbred line ELS-1 than in Yu87-1 from 25 to 30DAP. The phenotypes and the chlorophyll data showed that the leaf senescence of inbred line ELS-1 was earlier than the normal inbred line Yu87-1.

### Deep sequencing of small RNAs and their population distributions

**Fig. 2:**
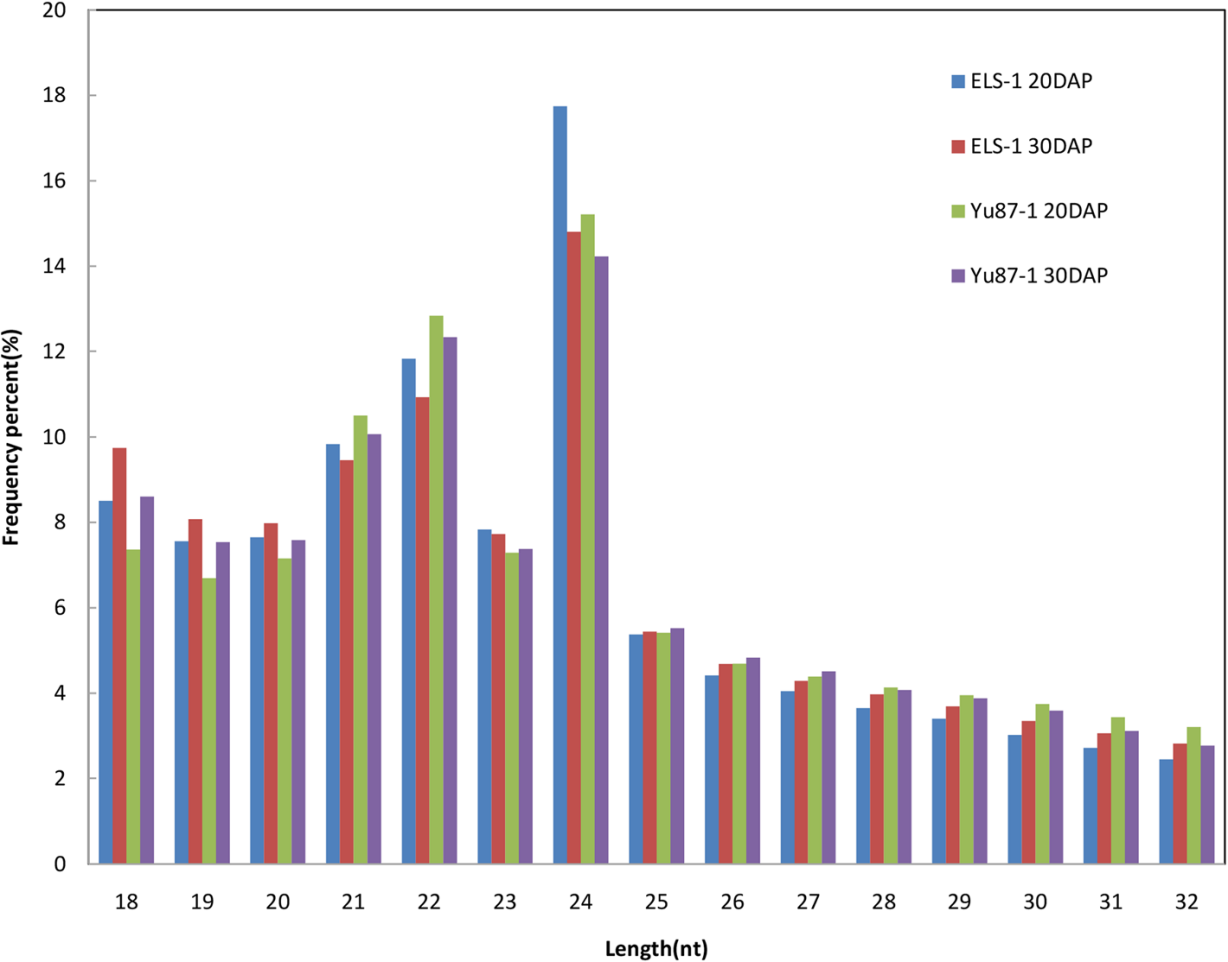
Length distribution and abundance of sRNAs in the samples. The 24-nt RNAs were the most abundant and more than half of the sRNAs ranged in length from 20 to 24nt (54.88 %, 50.89 %, 52.99 %, and 51.58 % for ELS-1 at 20DAP, ELS-1 at 30DAP, Yu87-1 at 20DAP, and Yu87-1 at 30DAP, respectively). miRNAs were 21–22nt, while siRNAs were 24nt

Four small RNA pools for the ear leaves collected at 20 and 30 DAP from the inbred lines ELS-1 and Yu87-1 were sequenced using the Illumina deep-sequencing technique to identify miRNAs involved in leaf senescence. In total, 56,346,694 reads were obtained from the four samples. After eliminating the other non-coding RNAs (rRNAs, tRNAs, snRNAs, and snoRNAs) and aligning the clean reads to the maize B73 genome (RefGen_v2), 215,506 candidate miRNA reads remained, ranging from 27,426 to 105,545 reads, with 53,876.5 average reads per timepoint (Additional file 1: Table S1). In all four samples, the 24-nt RNAs were the most abundant and more than half of the sRNA ranged in length from 20 to 24nt (Fig. 2).

### Known and novel miRNA identification during leaf senescence

The expression levels of miRNAs at 20 and 30 DAP in the inbred lines ELS-1 and Yu87-1 were analyzed and compared using the reads data from the deep sequencing. After filtering out inferior reads (RPKM < 5 in all samples), 80 miRNAs belonging to 26 miRNA families were identified (Additional file 1: Table S2). The expression patterns of 37 miRNAs were consistent between ELS-1 and Yu87-1 from 20 to 30DAP, with 15 miRNAs up-regulated and 22 miRNAs down-regulated. The other 44 miRNAs were expressed differently in the two inbred lines.

According to the analysis of the un-annotated small RNAs, 164 novel candidate miRNA loci were identified (Table 1). The majority of newly identified miRNAs were 20-23nt in length (Table 1), matching the size of the canonical miRNAs processed by Dicer-like proteins.

**Table 1:** Novel miRNAs abundance identified by small RNA libraries

novel miRNA	Length (nt)	sequence (5’--3’)	ELS-1 20DAP	ELS-1 30DAP	Yu87-1 20DAP	Yu87-1 30DAP
Predict-miR-1	21	CAGTGCATCCAAACAAGACCT	1.16	0.00	0.54	0.00
Predict-miR-2	22	GGAAATAATTTCTGATGACATA	0.00	0.00	1.63	2.26
Predict-miR-3	21	ATTAGGCTCGGGGACTATGGT	0.00	0.88	22.61	12.19
Predict-miR-4	22	TCTGTTTGGTTTTAGGGACTAA	1.54	0.00	3.08	0.00
Predict-miR-5	21	TCACTATCGCCGAATCTCGAA	1.93	0.00	0.54	0.00
Predict-miR-6	21	AAGTGCAGCCAAACAAGACCT	1.16	0.00	0.54	0.00
Predict-miR-7	22	ACTGGAATCCAACGGTCAGGAG	0.00	3.82	4.52	6.78
Predict-miR-8	21	TTTTAGCCCCTCCAATCTCCC	2.31	1.47	4.34	3.16
Predict-miR-9	23	TTCGAATTTCTGTCTATAATGCA	104.53	0.00	0.00	31.16
Predict-miR-10	21	CACTACAGATGAGACTAGGAG	1.93	1.18	0.00	0.00
Predict-miR-11	21	CGTCTTGTTTTTACTCCTATG	8.10	15.29	0.00	3.16
Predict-miR-12	21	GGGATTGGAGGAGCTAAAATC	0.00	0.29	0.72	0.00
Predict-miR-13	22	GAGGGACTAAAGACTAAAATAG	0.00	2.94	0.00	9.03
Predict-miR-14	21	GAGTTGATCTAGGTAATTATT	3.09	1.77	0.00	0.00
Predict-miR-15	23	TGCAGTAACGACAGTCAGAGCAG	0.00	0.00	1.99	1.36
Predict-miR-16	21	AAATACATTTTTAGGCCCTTG	0.00	0.88	0.00	3.61
Predict-miR-17	21	GTGAGAGAAGGGTATAATCAC	0.00	0.88	0.00	2.71
Predict-miR-18	22	CTTGGTAAAGTGACTCAACGTG	1.93	1.77	0.00	0.00
Predict-miR-19	21	GTATCCTTGTTCCCAAACAAG	0.00	1.18	2.89	0.00
Predict-miR-20	22	GATGGTTAACTGATGTACTCCA	0.00	0.00	0.54	2.26
Predict-miR-21	21	TAACGGGTAGAGAATCTTCTC	13.89	16.18	0.00	14.00
Predict-miR-22	20	CTTGAAGAGCAGCTAGGTCA	24.30	0.00	0.00	11.74
Predict-miR-23	20	TAGGTTAAGGCCTCGTTCGT	5.40	6.77	1.45	0.00
Predict-miR-24	23	AAATAATTTTTGATGACATACAG	0.00	0.00	1.63	2.26
Predict-miR-25	20	GCGAGAATGACGAAGAAAAT	0.00	0.00	2.53	9.03
Predict-miR-26	21	TAGTGATGCTATCAAGAATCA	0.39	0.29	0.00	0.00
Predict-miR-27	21	GTGATTATACCCTTCTCTCAC	0.00	0.88	0.00	1.81
Predict-miR-28	21	TTTTAGCCCCTCCAATCTCCT	0.00	0.00	4.34	3.61
Predict-miR-29	21	TGGTTCGTTGGATCTAGATTT	0.00	0.88	1.27	0.00
Predict-miR-30	21	ATGTAATTGAGACCCAAACAT	0.00	0.88	0.54	0.00
Predict-miR-31	21	TGGATTTTGATTGGATACATC	0.00	0.00	5.79	1.81
Predict-miR-32	20	AGCTGGAATAGCTCAGTTGG	46.29	23.53	3.26	9.49
Predict-miR-33	21	GGCATGCAAGTTCTCTAGCTA	0.00	1.18	1.63	0.00
Predict-miR-34	23	TCAGTCTGCCTTCTGTCTTTGAC	1.16	2.06	0.00	0.00
Predict-miR-35	21	CAAGGGCCTAAAAATGTATTT	0.00	0.88	0.00	3.61
Predict-miR-36	20	TGGTTCCCACTCATGACCCA	33.94	63.83	10.31	21.68
Predict-miR-37	21	AAGAGGATTGAAGGGACTAA	0.00	0.00	1.27	1.81
Predict-miR-38	22	TTCTTGTTTGGATATTTAGCTC	0.00	0.88	1.81	1.81
Predict-miR-39	21	ATGTGATTATACCCTTCTCTC	0.00	0.88	0.00	1.36
Predict-miR-40	21	TTATTCTGACATTTTGTTCTA	0.00	0.00	2.35	1.81
Predict-miR-41	21	GAGTTGATCTAGGTAACTAGT	2.31	1.77	0.00	0.00
Predict-miR-42	21	GAGCTCACCGGAACACCTCAT	0.00	0.00	0.72	1.36
Predict-miR-43	20	TTAAAGCTATATCATCACAT	0.00	2.06	0.00	1.81
Predict-miR-44	21	TGTTCGGTTGTGTGTGGATCG	1.93	0.00	2.71	8.58
Predict-miR-45	21	TTGTTAATGTTTGGAGTAGCG	11.96	0.00	0.00	6.78
Predict-miR-46	23	TGCCTCAGATGGACGCTTTGGAA	7.33	7.65	5.43	7.23
Predict-miR-47	23	TTCGAATTTTTGTCTATAATGCA	83.70	0.00	0.00	27.10
Predict-miR-48	22	TATGTATGGTCATGGTTGATGT	3.47	4.41	0.00	0.00
Predict-miR-49	21	GTTGGATTTTGTTTGGATGCA	0.00	0.00	5.07	1.81
Predict-miR-50	21	GAAACTGGTTTTTGCTGACAT	9.64	3.24	4.88	7.23
Predict-miR-51	21	TGCATCCAAACAAAATCCAAC	0.00	0.00	5.07	1.81
Predict-miR-52	22	CGGGCATGATAGACCAAGTCTA	10.80	0.00	0.00	11.29
Predict-miR-53	20	TGGGTCATGAGTGGGAACCA	33.94	63.83	10.31	21.68
Predict-miR-54	21	ATTTTAGCCTCTTCAATCCTC	0.00	0.00	34.73	29.81
Predict-miR-55	23	GCTAAAGTATATCAGGTCAAGGA	1.93	4.71	3.26	4.07
Predict-miR-56	21	CTACTTCCTTCGTTCCATAAA	0.00	0.00	0.91	3.16
Predict-miR-57	21	ATGCAGACGCAACCGAACAAG	0.77	0.00	0.18	0.00
Predict-miR-58	23	TGCTCAGCAGTCAGTAAGTCAGT	3.86	3.24	2.17	4.52
Predict-miR-59	23	TTCACTGTCAGAGATCTTCACGG	9.26	5.00	6.15	0.00
Predict-miR-60	21	GTCAATACTAGAGAATCCAAA	0.00	0.00	2.71	1.36
Predict-miR-61	21	ACTAGATTGGAGGGGCTAAAA	1.93	0.00	0.00	3.16
Predict-miR-62	21	CTGTTGGCTACCGTCGTCGAG	3.09	2.35	0.91	0.00
Predict-miR-63	23	TCCGGGCTTGTTTCGTAGAAATC	1.54	1.77	0.00	0.00
Predict-miR-64	21	AGTTAGATTTTGATTAGATGC	1.54	0.00	0.72	0.00
Predict-miR-65	21	GTCTGTAAATTGTTCTGCATA	27.39	19.12	38.35	31.16
Predict-miR-66	22	AGAGATTGTTATTTATAATGTT	0.00	0.00	1.63	1.36
Predict-miR-67	21	GCTCTCTCTGGTTCTCCCGAT	3.86	3.53	0.00	0.00
Predict-miR-68	20	AAGTTTAGGGATTTTTGGAG	1.54	0.00	0.00	7.68
Predict-miR-69	21	GGATCCAAACGCAACCGAACA	3.86	0.00	3.44	9.49
Predict-miR-70	22	AACATATGATGTTAACTAGGAG	0.00	0.00	0.54	1.81
Predict-miR-71	20	CTGGAATAGCTCAGTTGGTT	46.29	23.53	0.00	9.49
Predict-miR-72	21	GTTTGGGTCTCAATTACATCC	0.00	0.88	0.54	0.00
Predict-miR-73	20	TCGATCAGAACATCTGGCTT	1.54	1.77	0.00	0.00
Predict-miR-74	21	TAGCCTAAAACCGACGGGAAT	0.00	0.00	3.44	2.71
Predict-miR-75	21	TTTAACGGATAGAGAATCTTC	10.42	11.18	17.19	12.65
Predict-miR-76	21	GGGAGATTGGAGGGGCTAAAA	2.31	0.00	4.34	3.16
Predict-miR-77	20	AGCAGGAGGAAGGAGAGGAG	11.57	0.00	1.09	0.00
Predict-miR-78	22	AGAGATTTTTATTTATAGTGTT	0.00	0.00	1.81	1.36
Predict-miR-79	21	TGCATTCAATCAAAATCCAAC	0.00	0.00	5.07	1.81
Predict-miR-80	21	GTTAACGGATAGAGAATGTTC	0.00	0.00	2.53	0.45
Predict-miR-81	22	CTCCTGACCGTTGGATTCCAGT	1.16	3.82	4.52	6.78
Predict-miR-82	21	TTTATGGAACGAAGGAAGTAG	0.00	0.00	0.91	3.16
Predict-miR-83	21	CGATCCAAACGCAACCGAACA	3.86	0.00	3.26	9.49
Predict-miR-84	22	TAGACACAGATGGACCCTACAA	2.31	1.18	0.00	0.00
Predict-miR-85	20	TCATTTCGGCTCAGTCTTTC	16.59	7.65	0.00	9.94
Predict-miR-86	22	AGGTTAAGGACCTAGAAATGTA	0.00	1.18	2.71	2.71
Predict-miR-87	21	GAGGATTGAAGAGGCTAAAAT	0.00	0.00	34.55	29.81
Predict-miR-88	21	TTCTAGGTCTTGAACTTGTTA	2.70	1.77	0.00	0.00
Predict-miR-89	21	GCTTATTTACGTCGGTTTTAG	0.00	0.00	1.45	1.36
Predict-miR-90	21	GAGTTGATCTAGGTAATGAGT	2.31	1.77	0.00	0.00
Predict-miR-91	21	TTTTAGCCCCTCCAATCTAGT	1.93	1.18	3.26	3.16
Predict-miR-92	21	GTGATTATACCATTCTCTCAC	0.00	1.18	0.00	2.26
Predict-miR-93	21	ATGTCAGCAAAAACCAGTTTC	9.64	3.24	4.88	7.23
Predict-miR-94	22	GATTTTGAGCTTTACTGTTGGA	0.00	0.00	1.09	4.97
Predict-miR-95	21	AGGTCTTGTTTGGCTGCACTT	1.16	0.00	0.54	0.00
Predict-miR-96	20	TGTTGGTCAAAGTTTCAAAA	0.00	0.00	1.45	2.71
Predict-miR-97	21	TTTAGCCTCTACAATTCTCTC	0.00	0.00	29.31	25.74
Predict-miR-98	22	GGCTGACATGACCAAGTTCAAC	0.00	0.00	1.27	1.36
Predict-miR-99	20	TTGTGGTGATGAATTATGAA	5.79	15.88	12.84	21.68
Predict-miR-100	21	CGTTAGATTTTGATTGGATGT	1.16	0.00	1.45	0.00
Predict-miR-101	21	GTTGATCTAGATAATTAGTGA	2.31	1.77	0.00	0.00
Predict-miR-102	21	TGTTCGGTTGCGTTTGGATCC	0.00	0.00	3.44	9.49
Predict-miR-103	21	ACTCATTACCTAGATCAACTC	2.31	1.77	0.00	0.00
Predict-miR-104	21	ATCTAGATCTAACGGACTAAA	0.00	2.06	3.26	0.00
Predict-miR-105	23	TGCTCAGCAGTCAGTCAGTCAGT	3.09	0.00	1.81	3.61
Predict-miR-106	21	ATTAAATCTGGACCCTTCATC	5.01	2.35	0.00	2.71
Predict-miR-107	21	CCCGATGAATGTACATGATTT	0.00	8.53	3.98	4.97
Predict-miR-108	21	TGATCCAGACACAACCGAACA	0.00	9.41	0.00	13.55
Predict-miR-109	20	AACCAACTGAGCTATTCCAG	0.00	23.53	0.00	9.49
Predict-miR-110	21	ACTAGTTACCTAGATCAACTC	2.31	1.77	0.00	0.00
Predict-miR-111	21	ATGGATTTTAATTGGATGCAC	2.31	1.77	0.00	0.00
Predict-miR-112	21	TAGATTTTGATTGGATGCATC	0.00	0.00	5.79	2.26
Predict-miR-113	21	TGTTCGGTTGTGTCTGGATCA	7.33	9.41	9.05	13.55
Predict-miR-114	21	TTTTGTTAATGTTTGGAGTAG	11.96	15.00	0.00	6.78
Predict-miR-115	21	TGGATTGTATAATCTAGATAC	4.24	2.06	0.00	0.00
Predict-miR-116	20	GCTCTATCTTTAAGCAACTT	0.00	3.53	0.00	5.42
Predict-miR-117	21	AGGTCTTGTTTGGATGCACTG	1.16	0.00	0.54	0.00
Predict-miR-118	21	TTATGATATGTTACTCTACTA	0.00	0.00	5.25	4.07
Predict-miR-119	22	CGCCGAGTTCTGTAGCTAGAGC	133.46	0.00	86.11	0.00
Predict-miR-120	21	CCGAGGCGGGTTCTTCTCCTA	0.00	0.00	0.72	2.71
Predict-miR-121	21	GAGATTGGAGGGGCTAAAATC	2.70	0.00	4.34	3.61
Predict-miR-122	23	TTCGAATTCCTGTCTATAATGCA	74.45	0.00	1.63	0.00
Predict-miR-123	21	GAGTGGATTGTAGAGGCTAAA	0.00	0.00	30.93	25.29
Predict-miR-124	21	AGAACTACAATTTTCTAAGGG	0.00	0.00	1.27	1.36
Predict-miR-125	22	AAGGCTGACATGACCAAGTTCA	0.00	0.00	1.27	1.36
Predict-miR-126	21	TTTCTAGGTCTTGAACTTGTT	0.00	0.00	1.27	1.36
Predict-miR-127	21	CTCGACGACGGTAGCCAACAG	3.09	2.35	0.00	0.00
Predict-miR-128	23	TCCTTGACCTGATATACTTTAGC	1.93	4.71	3.26	4.07
Predict-miR-129	23	TATCCGGGCTTGTTTCGTAGAAA	1.54	1.77	0.00	0.00
Predict-miR-130	21	TATGCAGAACAATTTACAGAC	27.39	19.12	38.35	31.16
Predict-miR-131	20	GTTTAGGGATTTTTGGAGTT	1.54	0.00	0.00	7.68
Predict-miR-132	20	ATGTGATGATATAGCTTTAA	0.00	2.06	0.00	1.81
Predict-miR-133	23	ACTGACTTACTGACTGCTGAGCA	3.86	3.24	2.17	4.52
Predict-miR-134	21	TTTAGCCTCTACAATCCACTC	0.00	0.00	30.93	25.29
Predict-miR-135	21	CTTGTTCGGTTGCGCCTGGAT	6.17	8.24	6.33	4.97
Predict-miR-136	21	CTACTCCAAACATTAACAAAA	12.34	0.00	0.00	7.68
Predict-miR-137	21	TGTTCGGTTGCGTTTGGATCG	0.00	0.00	3.26	9.49
Predict-miR-138	21	GAGGATTGGAGAGGCTAAAAT	0.00	0.00	29.49	25.74
Predict-miR-139	23	TGGATCCTGGAGATAAGGCTGAA	33.94	28.82	62.59	0.00
Predict-miR-140	21	GAAAAGAACTTTCAAGAGAGA	5.01	55.88	0.00	22.13
Predict-miR-141	23	GGGCCTGATGCCACGTCTCTGTT	1.54	0.00	0.54	3.16
Predict-miR-142	21	TAGTAGAGTAACATATCATAA	0.00	0.00	5.25	4.07
Predict-miR-143	21	GTGGATTGTAGAGGCTAAAAT	0.00	0.00	30.93	25.29
Predict-miR-144	22	TTGGGGCGTAGCGTGAAAGCTG	1.16	0.00	0.00	1.36
Predict-miR-145	21	GAACATTCTCTATCCGTTAAC	0.00	0.00	2.53	0.45
Predict-miR-146	20	TTGAGAAAATTCATGAACGT	1.93	0.00	2.35	0.00
Predict-miR-147	21	GAAGATTCTCTATCCGTTAAA	10.42	11.18	17.19	12.65
Predict-miR-148	21	GAGAAGATTCTCTACCCGTTA	13.89	16.18	0.00	14.00
Predict-miR-149	22	AGGAGGTCCTGGACACATAAGT	0.00	1.18	5.43	4.07
Predict-miR-150	21	GATGAAGGGTCCAGATTTAAT	5.01	0.00	0.00	2.71
Predict-miR-151	21	TATCCCTCAGGATGCCACATC	0.00	0.00	1.27	2.26
Predict-miR-152	22	GATTTGGAGCTTTACTGTTGGA	0.00	0.00	1.45	5.87
Predict-miR-153	20	GGTGTAGTTGCTGATCCATA	0.00	2.94	0.54	0.00
Predict-miR-154	21	CTTGTTTGGGAACAAGGATAG	0.00	0.00	1.63	2.71
Predict-miR-155	21	GATTTTAGCCCCTCCAATCTC	2.70	0.00	4.52	3.61
Predict-miR-156	21	GTATCCTTGTTCCCAAACAAA	3.09	0.88	2.35	0.00
Predict-miR-157	22	TAAGAGATTGTTATTTATAGTG	0.00	0.00	1.81	1.36
Predict-miR-158	21	GTGAGAGAATGGTATAATCAC	0.00	1.18	0.00	2.26
Predict-miR-159	21	GGGATTAGAGGGGCTAAAATT	0.00	0.00	0.72	0.90
Predict-miR-160	21	ACCGTGGCTCCTGCTCCTGAT	0.00	0.00	9.05	2.71
Predict-miR-161	23	ATGCTAACACTGAACATTTTAGG	0.00	2.94	0.00	4.52
Predict-miR-162	20	GAGCCCAGCATTGTTATTTT	0.00	1.47	0.54	0.00
Predict-miR-163	23	GTGCTGTGATGAAGATGCTCAAC	92.57	252.07	98.59	168.01
Predict-miR-164	21	GAGAGAAGGGTATAATCACAT	0.00	0.88	0.00	1.36

### Differential expression of miRNAs between the two inbred lines

**Fig. 3:**
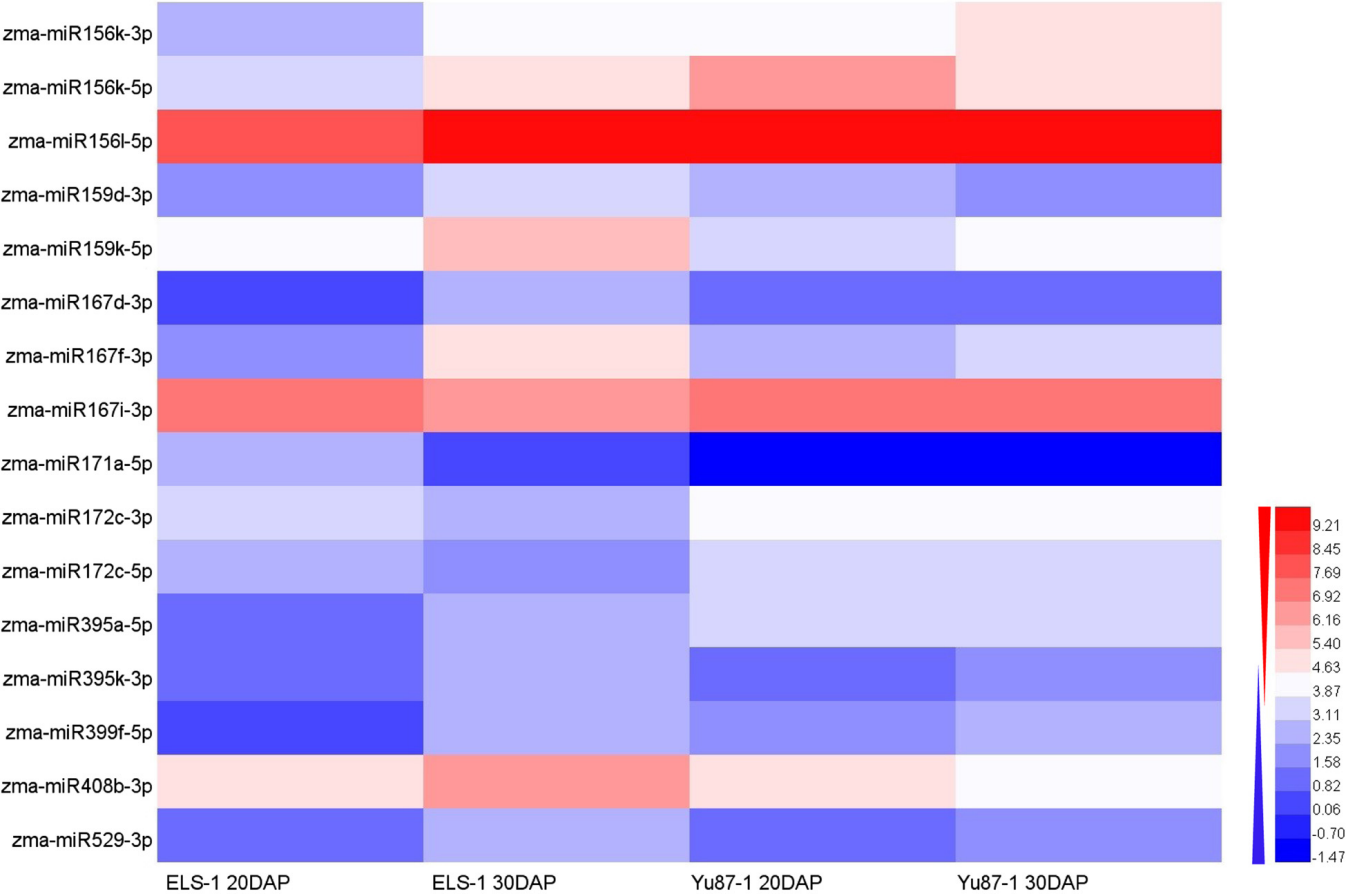
Expression levels of senescence-associated miRNAs in the leaves of inbred lines ELS-1 and Yu87-1. In total, 16 differentially expressed miRNAs were identified as candidate SA-miRNAs. Red indicates up-regulated gene expression and blue represents down-regulated. The scale is log_2_ (fold-change)

To investigate the miRNAs involved in leaf senescence from 20 to 30DAP, the expression profiles of miRNAs at 20 and 30DAP in the leaves of inbred line ELS-1 were analyzed. The stay-green line Yu87-1 was also analyzed as the control. The miRNAs in the leaves of the inbred line ELS-1 with expression levels, in log_2_-fold changes, were greater than 1.5 or less than −1.5, were considered potentially differentially expressed miRNAs that might participate in leaf senescence. Among those identified in the leaves of inbred line ELS-1, differentially expressed miRNAs whose |log_2_-fold change| was >1.5 in the leaves of inbred line Yu87-1 were removed, and 16 candidate miRNAs were finally identified in the leaves of inbred line ELS-1. These 16 differentially expressed miRNAs, belonging to nine miRNA families, zma-miR156, zma-miR159, zma-miR167, zma-miR171, zma-miR172, zma-miR395, zma-miR399, zma-miR408, and zma-miR529, were selected as candidate SA-miRNAs (Fig. 3). Among them, zma-miR167, zma-miR171,and zma-miR172 were down-regulated in both inbred lines, while zma-miR156, zma-miR395, zma-miR399, zma-miR408, and zma-miR529 were up-regulated in both inbred lines between 20 and 30DAP.

### Validation of miRNA expression profiling in leaf senescence

**Fig. 4:**
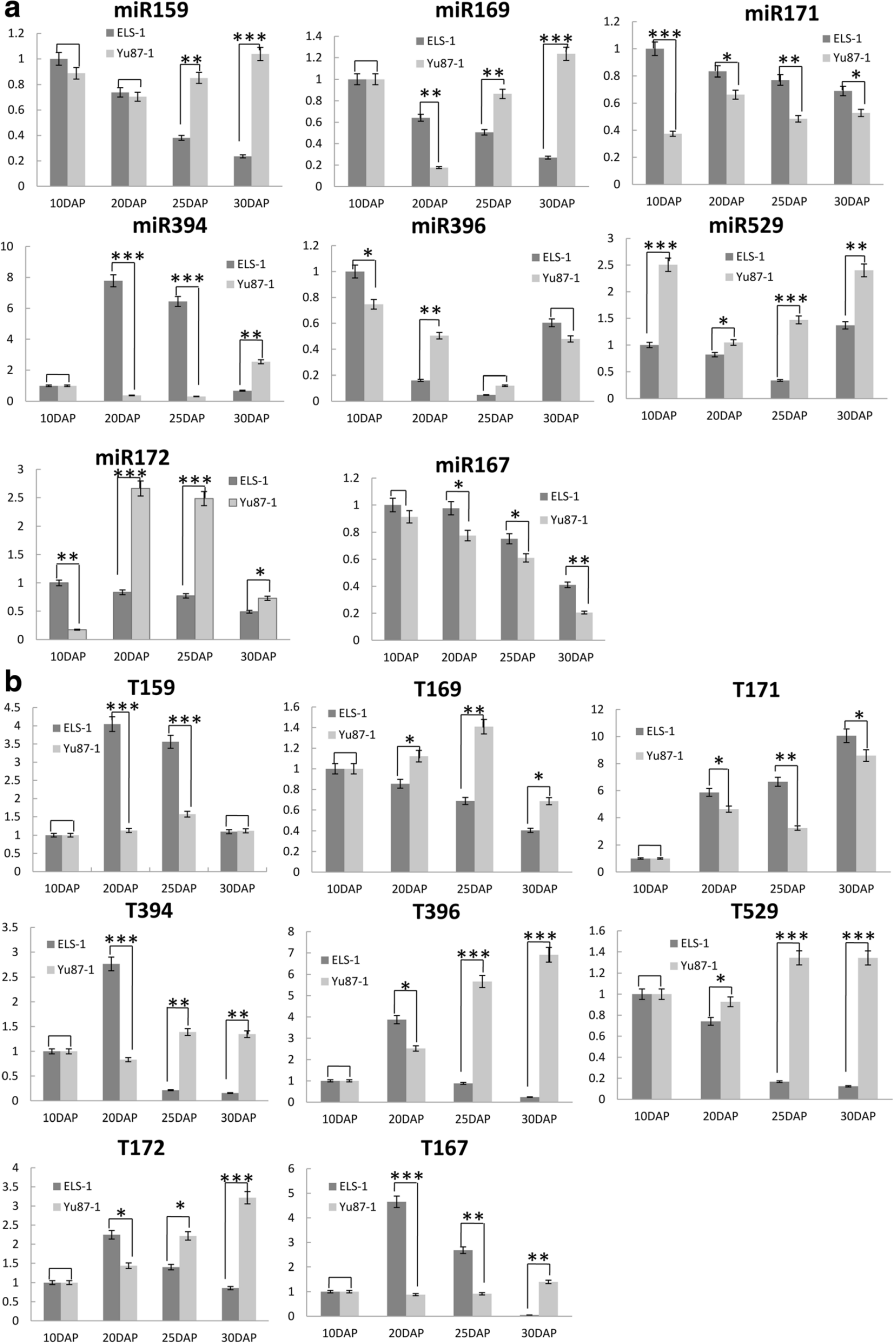
**a** Expression validation of the miRNAs. **b** Expression validation of the miRNAs’ target genes. Relative expression levels of the miRNAs in the two maize inbred lines at 10DAP, 20DAP, 25DAP, and 30DAP. *18SrRNA* is the reference gene. All of the qRT-PCR reactions were repeated three times per sample. T, target. The bars indicate the SE. Three biological and technical replicates were performed. *n* ≥ 3.^*^, *p* < 0.1;^**^,*p* < 0.01;^***^, *p* < 0.001

To validate the sequencing results and the miRNA expression patterns, the expression profiles of eight SA-miRNAs were validated by quantitative reverse transcription polymerase chain reaction (qRT-PCR) (Fig. 4). The differential expression levels of miRNAs from 20 to 30 DAP had similar trends by deep sequencing compared with the results of qRT-PCR.

Based on the profiles, almost all of the miRNAs were down-regulated in the ear leaves of the inbred line ELS-1, except miR396 and miR529, which had high expression levels on 30DAP and miR394, which had a low expression level on 10DAP. As for inbred line Yu87-1, the miRNA expression trend was more complex. Among the candidate miRNAs, miR159, miR169, miR394, and miR529 were up-regulated from 20 to 30DAP, and miR172, miR167, and miR171 were down-regulated from 20 to 30DAP.

Among the target genes profiled in the ear leaves of the inbred line ELS-1, the gene expression trends were opposite those of miR171, miR172, miR159, and miR396, suggesting that these miRNA target genes were subjected to a negative regulation by the miRNAs, most likely through a target cleavage pathway. In contrast, the expression trends of miR169 and miR394 were not opposite those of their target genes. A specific miRNA may have multiple targets and their expression levels may be subjected to transcriptional regulations, in addition to the post-transcriptional regulations by miRNAs. Similarly, the expression trends of miR159, miR169, and miR172 were negatively correlated with those of their target genes in the inbred line Yu87-1, while the expression levels of miR171 and miR529 were positively correlated with their target genes. Therefore, the effects of miRNAs and their targets were complicated and not always directly negatively correlated, as normally predicted.

### Target genes of miRNAs identified by degradome sequencing

**Fig. 5:**
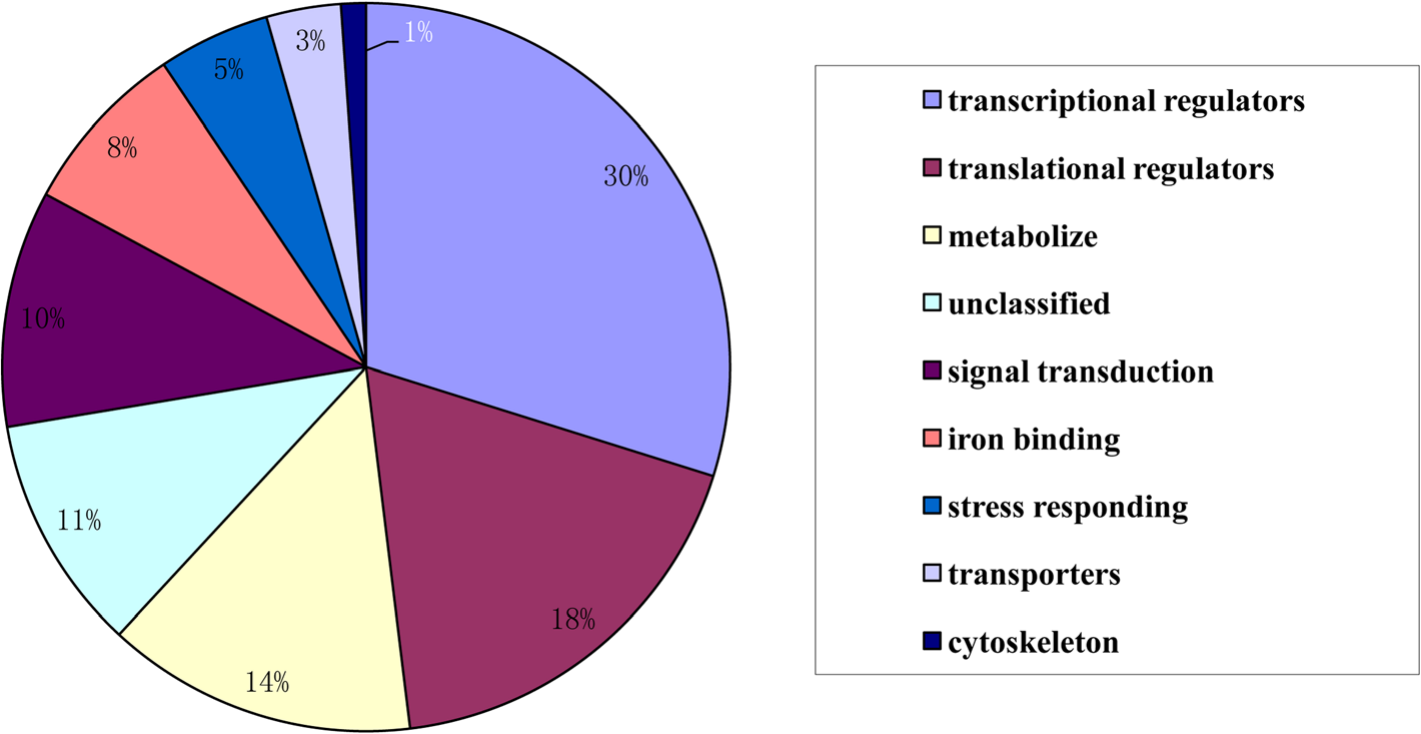
Functional classifications of the miRNAs’ target genes

To find the potential target genes for the different miRNAs, a genome-wide degradome sequencing analysis was performed using high-throughput degradome sequencing technology (Additional file 1: Table S3). Based on an analysis using the BLASTX algorithm, the targets of the candidate miRNAs were detected in the degradome libraries, and most of them, such as the SBP and MYB transcription factors, leucine-rich repeat receptor-like protein kinases (LRR-RLKs), AP2-like factor, zinc finger proteins, ubiquitin-conjugating enzyme E2,and beta-galactosidase, were involved in aging. A functional analysis indicated that 30 % of the target genes were classified as transcriptional regulators and 18 % were translational regulators (Fig. 5). Additionally, 14 %, 10 %, and 5 % of the genes were classified as metabolizes, signal transducers, and stress responders, respectively.

## Discussion

Maize is a major food and energy crop for human consumption, stock feed, and bioenergy production. As an integral part of plant development, leaf senescence involves a whole-plant senescence process in which minerals and carbohydrates are mobilized and translocated to the storage organs from the vegetative plant parts [24]. Early leaf senescence caused by intrinsic or environmental factors results in a photosynthetic decline and precocious whole-plant aging [25]. Because the relationship between senescence and crop productivity is complex [26], and hybrid maize plants always have a short grain-filling period, early leaf senescence seriously affects grain yields. In the leaf senescence process, the most obvious phenotypic characteristic was leaf yellowing, which results from the preferential breakdown of chlorophyll and chloroplasts [27]. Compared with the inbred line Yu87-1, the inbred line ESL-1 showed a faster decline in chlorophyll *a* and *b* levels at 25DAP and later, which is similar to the levels seen in lines in which pollination is prevented [28], indicating that the source/sink ratio might cause the leaf senescence in the inbred line ESL-1.

Previous research considered leaf senescence as a complicated and highly regulated developmental process, and many SAGs were identified in Arabidopsis, wheat, rice, and maize [4]. In addition to the SAGs, several other genetic regulatory mechanisms involved in leaf senescence have been identified. The miRNA pathway was the last mechanism that was discovered to be responsible for leaf senescence and its regulatory network. MiR164 and miR319 were identified to target *ORE1* and *TCP* transcription factors, and both are negative regulators of leaf aging [17–22]. In this study, 16miRNAs were identified as being differentially expressed between the early leaf senescence line ELS-1 and the normal green-stayed inbred line Yu87-1. The target genes of the leaf SA-miRNAs were identified by degradome sequencing and categorized as being involved in transcriptional regulation and other biological processes.

The receptor-like kinases (RLKs) are cell surface receptors, and most plant RLKs have extracellular receptor, single-pass transmembrane, and intracellular kinase domains [29]. Several RLKs are involved in a diverse range of developmental and stress signal transduction pathways, such as Brassica SRK in self-incompatibility [30] and rice *Xa21* in resistance to bacterial pathogens [31]. The transcription levels of *PvSARK* in bean and *AtSIAK* in Arabidopsis increased in senescent leaves [32, 33]. The *rlpk2* gene, cloned in an artificially induced senescent soybean mutant, encodes a LRR-RLK protein [34]. The RNA interference-mediated knock down of *rlpk2* dramatically retarded both the natural and nutrient deficiency-induced leaf senescence in transgenic soybean, and its leaves showed higher chlorophyll contents and a deeper green color. These results indicated that LRR-RLKs might be involved in the regulation of chloroplast development and chlorophyll accumulation. In the present study, two differentially expressed miRNAs, zma-miR167d and zma-miR171a, which were predicted to target genes encoding LRR-RLKs protein, were identified as down-regulated in the leaves of inbred lines ELS-1 and Yu87-1. At the late stage of leaf development (30DAP), the expression levels of zma-miR167d and zma-miR171a were higher in the leaves of the inbred line ELS-1 than in Yu87-1. Higher levels of zma-miR167d and zma-miR171a might decrease the expression of LRR-RLKs, which was predicted to promote leaf senescence.

Transcription factors often act as switches causing differential gene expression levels by binding to specific *cis* elements in the promoters of the target genes, which results in their activation and/or suppression. Using a leaf senescence EST library, Guo [7] collected ~130 senescence-associated transcription factors, which belonged to the *NAC*, *WRKY*, *AP2*, *MYB*, zinc finger proteins, and *bZIP* families. *AP2* is involved in fruit ripening and plant senescence regulation in an ethylene/ABA-dependent way [35, 36]. Osa-miR172a, osa-miR172c, and osa-miR172d were identified as targeting genes of AP2-like factors in the leaves of rice [23]. High levels of osa-miR172a, osa-miR172c, and osa-miR172d depressed the expression levels of AP2-like factor genes, which retarded leaf senescence. In correlation to this study, the expression levels of zma-miR172c in the leaves of the inbred line ELS-1 were lower than in the inbred line Yu87-1 during the late stage of leaf development (20-30DAP). Additionally, zma-miR172c negatively regulated its target genes, which encoded AP2 proteins, as identified through degradome sequencing (Fig. 4). Based on the phenotypes of ELS-1 and Yu87-1 observed in this study, we concluded that miRNA-mediated leaf senescence, as an additional mechanism, exists in maize.

*MYB* genes were first identified as oncogenes derived from retroviruses in animal cells that encoded transcription factor proteins and shared the conserved MYB DNA-binding domain [37]. In plants, MYB proteins are divided into four classes depending on the number of MYB DNA-binding repeats: MYB-related, R2R3-MYB, R1R2R3-MYB, and atypical MYB family [38]. In the past decade, many researchers have focused on the roles of MYB transcription factors in growth control, hormone signaling, and stress response, and a few studies implicated MYB transcription factors in the regulation of leaf senescence. *Mybh-1*, a *mybh* mutant line, exhibits a delayed-senescence phenotype, while over expressing *MYBH* causes premature leaf senescence and enhances the expression of leaf senescence marker genes [39]. The AUX-responsive phenotype is enhanced in MYBH overexpression lines and was reduced in a *mybh* knockout line, suggesting that MYBH mediated leaf senescence by AUX homeostasis. The ectopic expression of a MYB-related transcription factor, *AtMYBL*, triggered early leaf senescence in *A. thaliana* [29]. Guo and Gan [40] found that *AtMYB2* regulates whole-plant senescence by inhibiting cytokinin-mediated branching during the late stages of Arabidopsis development. In the present study, zma-miR159d was identified as a differentially expressed miRNA, which was predicted to target genes encoding the MYB transcription factor by degradome sequencing. The expression level of zma-miR159d was down-regulated in the leaves of the inbred line ELS-1, while it had a higher expression level in the leaves of the inbred line Yu87-1 during the 25-30DAP (Fig. 4). The different expression profiles might be one of the reasons for the rapid decrease in the chlorophyll content that induced the earlier leaf senescence in the inbred line ELS-1.

The gene ontology of the SA-miRNAs target genes, identified by degradome sequencing, have been analyzed. Unlike the major targets of the conserved miRNAs, which were mainly involved in transcriptional and translational regulation, the target genes of SA-miRNAs had distinct functions. In the present study, the newly predicted miRNAs, miR-36, miR-131, and miR-11, were characterized as targeting the gene transcripts encoding *psbA* and 30S ribosomal proteins for regulation. *psbA* encodes a photosystem Q (B) protein involved in the photosynthetic electron transport in photosystem II, and leaf senescence was promoted in tobacco homoplasmic *psbA* deletion mutants [41]. Ribosomal proteins in plants regulate cell growth and death [42]. Individual ribosomal proteins, and changes in their expression levels, participate in and modulate a wide variety of activities associated with cell growth and death. The 30S ribosomal protein was identified via a proteome study in maize leaves and was characterized as a nitrogen-related senescence protein [43]. Combined with the tissue-specific expression model and the target specificity, it is highly possible that the novel miRNAs were transiently and spatially expressed to fulfill the special functions needed.

**Fig. 6:**
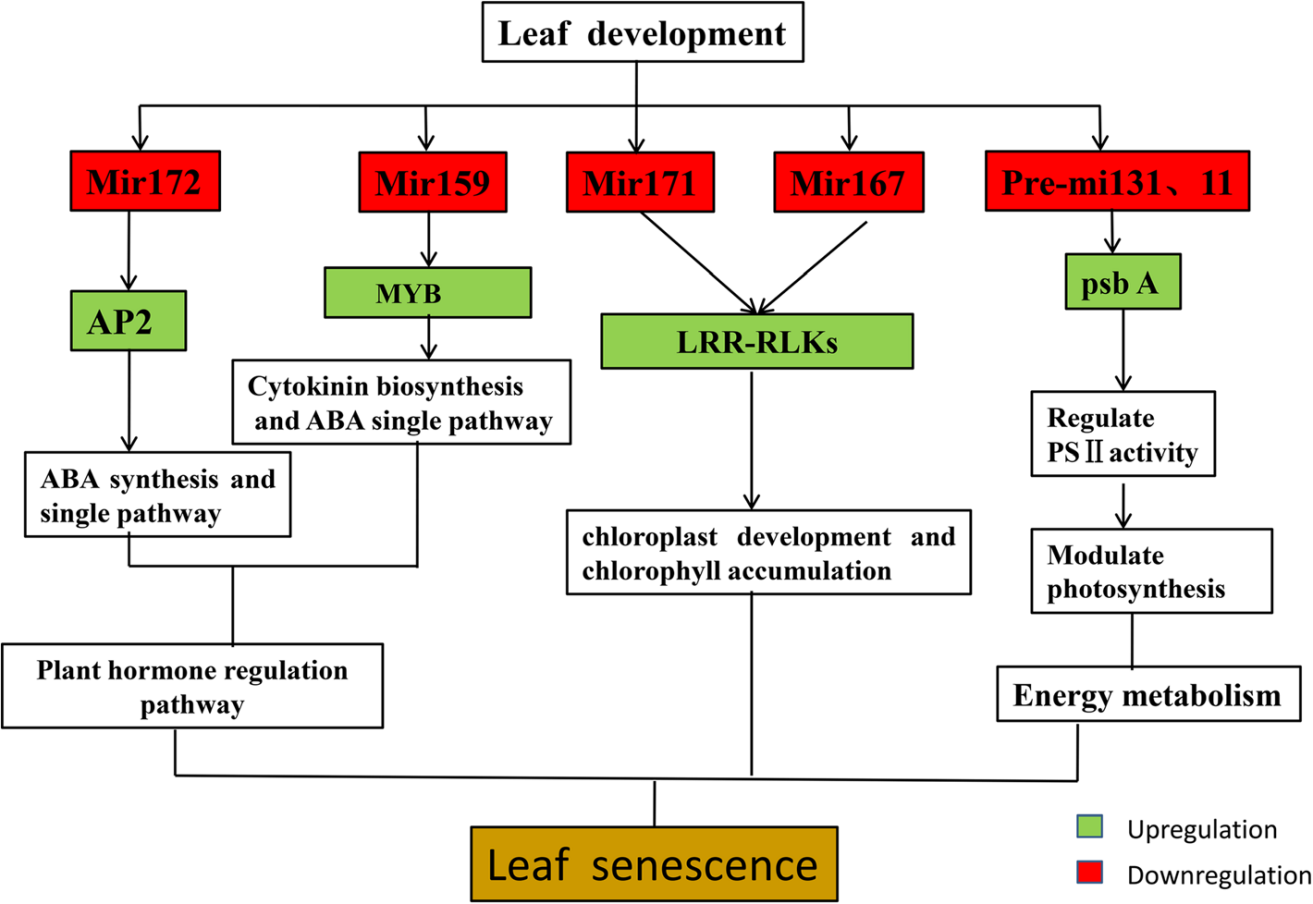
The miRNA-dependent gene expression network. The miRNA-dependent leaf senescence pathway was obtained based on the differential expression levels and their potential functions. Green indicates up-regulation with leaf senescence and red indicates down-regulation

In this study, six miRNAs regulating nodes, miR172, miR159, miR171, miR167, pre-mir131, and pre-mir11, were highlighted as key regulators of leaf senescence because of their differential expression profiles between ELS-1 and Yu87-1, and a candidate miRNA-dependent leaf senescence pathway was established based on the differentially expressed miRNAs and their potential functions discussed (Fig. 6). The miRNAs associated with leaf senescence in this study mainly functioned through three pathways: plant hormone regulation, chlorophyll metabolism, and energy metabolism. Even though the mechanisms underlying these pathways and their effects on leaf senescence are not clean, our data provide valuable information for understanding the roles of miRNAs in leaf senescence regulation.

## Conclusions

The inbred line ELS-1 in maize is an early leaf-senescence line with rapidly declining chlorophyll *a* and *b* levels at 25DAP and later. Using deep RNA-sequencing and degradome sequencing, 16 differentially expressed miRNAs were identified between the two inbred lines in this study. These differentially expressed miRNAs were candidate SA-miRNAs with potential roles in the leaf senescence regulatory network. MiRNA analyses indicated that these SA-miRNAs mainly targeted the SBP and MYB transcription factors, LRR-RLKs, AP2-like factor, zinc finger proteins, ubiquitin-conjugating enzyme E2, and beta-galactosidase. Their targets were speculated to regulate leaf senescence mainly by controlling chlorophyll degradation and transcription factor-mediated phytohormone pathways.

## Methods

### Plant materials

To investigate miRNAs and their target transcripts associated with maize leaf senescence, the inbred line ELS-1, which was an early leaf senescence inbred line found during maize breeding, and the elite maize inbred line Yu87-1, as the control, were used in this study. The inbred line ELS-1 was selected from an offspring of Vietnamese germplasm during recycled line breeding, and the inbred line Yu87-1 was selected from an American germplasm [44]. In total, 120 plants of each inbred line were planted in the summer of 2012 on the farms of Henan Agricultural University (Zhengzhou, China; E113°42’, N34°48’),where the average temperature is 14.3 °C and the average rainfall is 640.9 mm per year. In the field, the whole plants of the two inbred lines, including all of the leaves, were harvested in the morning from 20 to 30DAP. The middle part of the ear leaves were then carefully harvested using a knife before being stored in a −80 °C freezer. Each sample had three biological repeats.

### Determining the chlorophyll content

To characterize the changing chlorophyll content, the middle part of the ear leaf was sampled, with three biological repeats. For each sample, 0.2g leaves were collected and cut into pieces before soaking with 20mL mixed extract buffer (ethonal:acetone:H_2_O = 4.5:4.5:1). The chlorophyll *a*, chlorophyll *b*, and total chlorophyll contents in the leaves of inbred lines ELS-1 and Yu87-1 were detected and analyzed using the methods of Shen [45] with a modification. The mixed extract with leaves was stored in the dark at room temperature and shaken every 2h. After 24h, it was diluted with extraction buffer to 20mL, and the absorption values were measured at 645nm and 663nm. Each measurement was repeated three times and each sample had three replicate. Data were analyzed statistically using variance measurements.

### miRNA deep sequencing

Total RNA from each leaf tissue was extracted immediately with TRIzol reagent (Invitrogen, Carlsbad, CA, USA) following the manufacturer’s protocol. Approximately 20μg of total RNA was extracted. Considering the economic cost and representativeness of the samples, biological repeats were equally mixed for deep sequencing. Then, the mixture was purified for Illumina deep sequencing (YQYK-Biotech, Beijing, China). Small RNAs were ligated to a 5’ adaptor and a 3’ adaptor sequentially using T4 RNA Ligase 1 and T4 RNA Ligase 2 (truncated), respectively. They were then amplified using reverse-transcription polymerase chain reaction (RT-PCR), enriched by polyethylene glycol precipitation, and separated on a 15 % denaturing polyacrylamide gel. Small RNAs of 18–32nt were gel purified and directly used for sequencing.

### miRNA data analysis

For the reported maize miRNAs, the raw data from Illumina sequencing were pre-processed to filter out adaptor sequences and low-quality tags, and the appropriate 18-32nt tags of sRNA clean reads were obtained. The clean reads were mapped to the maize B73 genome and the Rhfm (11.0) database (http://rfam.sanger.ac.uk). The rRNA, tRNA, snRNA, snoRNA, and Cis-regRNAs were removed from the clean reads. Conserved miRNAs, which had been previously reported in plant species, were annotated by comparison with the maize miRNAs in miRBase (http://www.mirbase.org/). The reads per million (RPM) and the fold changes were used to measure the relative expression levels of each conserved miRNA, and to avoid sequencing errors, a 5 RPM value (in at least one sample) was set as the lowest threshold. The miRNAs that showed expression level differences of more than 1.5-fold between the two inbred lines were treated as candidate miRNAs involved in the determination of leaf senescence.

Based on the biological characteristics of the miRNAs, novel miRNAs were predicted using Mireap (http://sourceforge.net/projects/mireap/) from the un-annotated small RNA that were mapped to the maize genome. The criteria for selecting potential small RNA sequences followed those of Ding [46].

### Genome-wide degradome sequencing

The total RNA quantity and purity were analyzed using a Bioanalyzer 2100 and RNA 6000 n LabChip Kit (Agilent Technologies, Palo Alto, CA, USA) with PIN > 7.0. Approximately 20μg of total RNA of mixed samples from each time point for both lines were used to prepare the degradome library. The method differed considerably from past efforts [47, 48] and followed that of Ma [49] with some modifications: (1) Approximately 150ng of poly(A)^+^ RNA was used as the input RNA and annealing used biotinylated random primers; (2) The strapavid in capture of RNA fragments used biotinylated random primers; (3) 5’ adaptor ligation to only those RNAs containing 5’-monophosphates; (4) Libraries were sequenced using the 5’ adapter only, resulting in the sequencing of the first 36 nucleotides of the inserts, which represented the 5’ ends of the original RNAs. Single-end sequencing (36bp) was performed on the Illumina Hiseq2500 following the recommended protocol.

Raw reads were obtained using Illumina’s Pipeline v1.5 software, followed by the sequencing image analysis using the Pipeline Firecrest Module. The extracted sequencing reads were used in the following data analyses. CleaveLand3.0 was used for analyzing the generated sequencing data (Additional file 2: Figure S1).

### Validation of candidate miRNAs and target genes

Eight miRNAs and their target genes were selected to validate the sequencing results using real-time RT-PCR analysis. The total RNA of leaves was extracted using TRIzol (Takara, Dalian, China) following the manufacturer’s instructions. The RT reaction was executed following the instructions of the SYBR Prime Script miRNA RT-PCR Kit (Takara, Dalian, China). The qRT-PCR reaction was carried out by SYBR green II (Takara, Dalian, China) on an IQ5 real-time PCR machine (Bio-Rad, Hercules, CA, USA) following efforts of a previous protocol [50] with some modifications: (1) a maize house-keeping gene, *18SrRNA*, served as the internal control for real-time RT-PCR reactions; and (2) the annealing temperature was changed into 58 °C. The 2^–ΔΔCt^ method was used to calculate the relative expression levels. Each real-time qRT-PCR reaction was performed in sampling and technical triplicates. The PCR primers for the selected miRNAs and their target genes are listed in Additional file 1: Table S4. An analysis of variance was performed on the qRT-PCR data using the sampling and technical triplicates.

### Availability of supporting data

All of the sequencing data generated in this study is available from the SRA-Archive (http://www.ncbi.nlm.nih.gov/sra) under the study accession numbers SRX1445134, SRX1445135, SRX1445136, and SRX1445137, representing miRNA sequence data of ELS-1 on 20DAP and 30DAP, and Yu87-1 on 20DAP and 30DAP, respectively. The accession number of the degradome sequence data is SRX1433266.

## Additional files

Additional file 1: Table S1. Summary of small RNA classes in the samples. **Table S2**. Expression abundance of the known miRNAs. **Table S3.** Regions of the target genes identified by degradome sequencing. **Table S4.** Primers used in miRNA and target gene validation. (DOCX 61 kb) Additional file 2: Figure S1. T-plot of miRNA target genes. Five different categories of T-plots are shown. The degradome tag distributions along the target mRNA sequence are exhibited. The red line represents the sliced target transcripts. (TIF 4679 kb)

## Abbreviations

miRNAs: MicroRNAs
SA-miRNAs: aenescence-associated miRNAs
ELS: early leaf senescence
DAP: days after pollination
SAGs: senescence-associated genes
NAC: NAM, ATAF, and CUC
SBP: SQUAMOSA promoter-binding protein
AP2: APETALA2
AUX: auxin
ABA: abscisic acid
JA: jasmonic acid
SA: salicylic acid
EIN3: ETHYLENE INSENSITIVE3
Nt: nucleotide
CCGs: chlorophyll catabolic genes
TCP: TEOSINTE BRANCHED/CYCLOIDEA/PCF
LOX2: lipoxygenase 2
qRT-PCR: quantitative reverse transcription polymerase chain reaction
LRR-RLK: Leucine-rich repeat receptor-like protein kinase
RLKs: receptor-like kinases
RT-PCR: reverse transcription polymerase chain reaction
RPM: reads per million
