# Supplementary material for: microRNA-dependent gene regulatory networks in maize leaf senescence
Source: BMC Plant Biol. 2016 Mar 22;16:73. doi: 10.1186/s12870-016-0755-y (PMC4802599; doi:10.1186/s12870-016-0755-y)
Supplement: Additional file 1: Table S1. — Summary of small RNA classes in the samples. Table S2. Expression abundance of the known miRNAs. Table S3. Regions of the target genes identified by degradome sequencing. Table S4. Primers used in miRNA and target gene validation. (DOCX 61 kb) [file 12870_2016_755_MOESM1_ESM.docx]

Supplemental Table 1. The summary of small RNA classes in the samples.

|  | ELS-1 20DAP | | ELS-1 30DAP | | Yu87-1 20DAP | | Yu87-1 30DAP | |
| --- | --- | --- | --- | --- | --- | --- | --- | --- |
|  | Unique | Reads | Unique | Reads | Unique | Reads | Unique | Reads |
| map | 934502 | 5185062 | 1512378 | 6799846 | 2E+06 | 11055800 | 793404 | 4428270 |
| anno | 125178 | 1287433 | 172734 | 1637142 | 172734 | 3184402 | 121482 | 1194037 |
| rRNA | 52839 | 430172 | 66460 | 598126 | 66460 | 835002 | 47069 | 384781 |
| tRNA | 24633 | 614872 | 31292 | 635565 | 31292 | 1844424 | 25659 | 575551 |
| snRNA | 23124 | 79825 | 38565 | 159859 | 38565 | 158761 | 23420 | 79403 |
| miRNA | 3687 | 27426 | 5627 | 45541 | 5627 | 105545 | 3883 | 36994 |
| other | 20895 | 135138 | 30790 | 198051 | 30790 | 240670 | 21451 | 117308 |
| No_annotation | 809324 | 3897629 | 1339644 | 5162704 | 1E+06 | 7871398 | 671922 | 3234233 |

Supplemental Table 2. Expressional abundance of the known miRNAs.

| Family | Known-miR | ELS-1 20DAP | ELS-1 30DAP | Yu87-1 20DAP | Yu87-1 30DAP |
| --- | --- | --- | --- | --- | --- |
| zma-miR1432 | zma-miR1432a | 8.10 | 6.77 | 5.97 | － |
|  | zma-miR1432b | 63.26 | 57.35 | 114.15 | 83.10 |
| zma-miR156 | zma-miR156e | 27.00 | 14.71 | 31.48 | 14.00 |
|  | zma-miR156g | 18.13 | 26.77 | 22.97 | 36.13 |
|  | zma-miR156k | 8.10 | 17.65 | 19.90 | 26.20 |
|  | zma-miR156l | 12.34 | 34.12 | 77.97 | 39.75 |
|  | zma-miR156m | 329.02 | 702.96 | 1005.08 | 778.63 |
| zma-miR159 | zma-miR159a | 14.66 | 18.82 | 29.31 | 45.62 |
|  | zma-miR159d | 4.63 | 12.06 | 5.07 | 4.52 |
|  | zma-miR159f | 18.71 | 13.24 | 17.01 | 27.32 |
|  | zma-miR159k | 1402.11 | 1847.40 | 2759.82 | 2687.28 |
|  | zma-miR159l | 24.30 | 60.30 | 12.48 | 20.32 |
| zma-miR160 | zma-miR160a | 16.59 | 20.59 | 47.22 | 28.00 |
| zma-miR162 | zma-miR162b | 128.06 | 92.36 | 240.42 | 108.39 |
| zma-miR164 | zma-miR164a | 1.93 | 2.65 | 5.25 | 4.52 |
|  | zma-miR164e | 1.16 | 2.94 | 52.28 | 14.45 |
| zma-miR166 | zma-miR166a | 307.04 | 376.19 | 714.74 | 462.03 |
|  | zma-miR166c | 9.64 | 19.12 | 23.34 | 31.16 |
|  | zma-miR166d | 224.88 | 255.59 | 335.57 | 219.50 |
|  | zma-miR166h | 8.10 | 8.24 | 11.58 | 7.23 |
|  | zma-miR166j | 14.66 | 17.06 | 4.52 | 5.87 |
|  | zma-miR166m | 115.72 | 114.71 | 161.00 | 102.07 |
|  | zma-miR166n | 1.93 | 8.53 | 5.25 | 1.36 |
| zma-miR167 | zma-miR167c | 72.13 | 61.18 | 99.86 | 73.17 |
|  | zma-miR167d | 1.54 | 6.18 | 2.17 | 2.26 |
|  | zma-miR167e | 27.00 | 19.41 | 18.45 | 23.94 |
|  | zma-miR167f | 4.63 | 31.03 | 6.78 | 11.97 |
|  | zma-miR167g | 43.20 | 42.94 | 10.31 | 8.58 |
|  | zma-miR167i | 182.45 | 86.77 | 187.41 | 127.36 |
|  | zma-miR167j | 173.96 | 169.12 | 278.59 | 281.37 |
|  | zma-miR167k | 882.92 | 957.67 | 1497.68 | 1116.46 |
| zma-miR168 | zma-miR168a | 32.40 | 24.41 | 24.78 | 37.94 |
|  | zma-miR168b | 13.50 | 15.29 | 28.04 | 29.81 |
|  | zma-miR168d | 74.45 | 95.59 | 138.03 | 128.72 |
| zma-miR169 | zma-miR169b | 28.16 | 28.24 | 28.22 | 19.42 |
|  | zma-miR169q | 0.77 | 0.59 | 7.24 | 9.94 |
| zma-miR171 | zma-miR171a | 5.40 | 1.47 | 0.36 | 0.45 |
|  | zma-miR171g | 4.24 | 5.29 | 10.49 | 10.39 |
|  | zma-miR171h | 3.86 | 5.59 | 6.15 | 6.32 |
|  | zma-miR171i | 4.63 | 7.06 | 3.08 | 4.07 |
|  | zma-miR171k | 4.63 | 6.18 | 10.49 | 6.32 |
| zma-miR172 | zma-miR172b | 9.64 | 8.53 | 8.50 | 7.68 |
|  | zma-miR172c | 11.96 | 5.59 | 21.71 | 15.81 |
|  | zma-miR172d | 7.33 | 3.24 | 8.86 | 10.84 |
|  | zma-miR172e | 28.16 | 25.30 | 75.44 | 46.97 |
| zma-miR2275 | zma-miR2275b | 0.39 | 5.29 | 0.54 | 3.16 |
|  | zma-miR2275d | 5.59 | 5.29 | 1.45 | 3.61 |
| zma-miR319 | zma-miR319c | 10.42 | 9.12 | 5.97 | 11.74 |
| zma-miR393 | zma-miR393c | 24.69 | 10.29 | 26.95 | 12.19 |
|  | zma-miR393d | 10.03 | 9.71 | 18.09 | 4.97 |
| zma-miR394 | zma-miR394b | 6.94 | 9.12 | 14.47 | 6.78 |
| zma-miR395 | zma-miR395a | 2.70 | 6.18 | 9.23 | 9.49 |
|  | zma-miR395k | 2.31 | 5.59 | 2.71 | 3.16 |
|  | zma-miR395l | 48.60 | 60.59 | 45.77 | 88.97 |
|  | zma-miR395m | 10.42 | 8.53 | 1.27 | 4.07 |
|  | zma-miR395o | 3.09 | 4.41 | 5.79 | 4.97 |
|  | zma-miR395p | 2.70 | 1.77 | 13.75 | 7.68 |
| zma-miR396 | zma-miR396a | 18.90 | 23.24 | 55.90 | 49.23 |
|  | zma-miR396c | 47.06 | 47.94 | 90.09 | 91.68 |
|  | zma-miR396d | 134.23 | 198.53 | 277.14 | 282.73 |
|  | zma-miR396f | 7.33 | 5.29 | 1.63 | 2.71 |
|  | zma-miR396i | 24.30 | 22.06 | 58.43 | 42.46 |
|  | zma-miR396g | 20.44 | 20.30 | 40.34 | 31.62 |
|  | zma-miR396h | 6.94 | 10.00 | 18.45 | 18.07 |
| zma-miR397 | zma-miR397b | 3.86 | 13.53 | 52.10 | 9.49 |
| zma-miR398 | zma-miR398a | 3.47 | 5.59 | 3.80 | 3.16 |
|  | zma-miR398b | 6.94 | 42.65 | 103.84 | 25.74 |
|  | zma-miR398f | 4.63 | 12.94 | 60.24 | 7.23 |
| zma-miR399 | zma-miR399b | 3.86 | 5.29 | 0.91 | 2.26 |
|  | zma-miR399e | 12.73 | 21.77 | 5.79 | 14.00 |
|  | zma-miR399f | 1.54 | 5.59 | 3.80 | 5.42 |
|  | zma-miR399j | 4.63 | 10.00 | 19.00 | 9.49 |
| zma-miR408 | zma-miR408b | 33.56 | 99.71 | 28.58 | 18.97 |
|  | zma-miR408e | 0.77 | 5.00 | 1.27 | 3.61 |
| zma-miR444 | zma-miR444b | 103.37 | 96.47 | 134.41 | 143.17 |
| zma-miR528 | zma-miR528b | 5.79 | 5.00 | 7.24 | 7.68 |
|  | zma-miR528e | 6.56 | 11.18 | 26.95 | 9.94 |
| zma-miR529 | zma-miR529a | 2.31 | 8.53 | 2.71 | 4.07 |
| zma-miR827 | zma-miR827b | 176.28 | 320.01 | 121.57 | 445.32 |
|  | zma-miR827e | 32.79 | 63.53 | 17.37 | 96.20 |

Note: “– ” means no read was found.

Supplement Table 3. Part of the targets genes identified by degradome sequencing.

| SmallRNA | Transcript | Alignment Score | Alignment Range | Cleavage Site | Category |
| --- | --- | --- | --- | --- | --- |
| Predict-miR-102 | GRMZM2G100246_T01 | 2.5 | 1643-1663 | 1654 | 4 |
| Predict-miR-109 | GRMZM5G806488_T01 | 4 | 5169-5189 | 5180 | 4 |
| Predict-miR-11 | GRMZM2G144558_T01 | 0.5 | 299-319 | 310 | 4 |
|  | GRMZM2G474534_T02 | 0.5 | 535-555 | 546 | 4 |
|  | GRMZM5G844628_T01 | 0.5 | 1075-1095 | 1086 | 4 |
|  | GRMZM2G158333_T01 | 0.5 | 607-627 | 618 | 4 |
|  | GRMZM2G450705_T02 | 0.5 | 1891-1911 | 1902 | 4 |
|  | GRMZM2G474534_T01 | 0.5 | 2119-2139 | 2130 | 4 |
|  | GRMZM2G383052_T01 | 0.5 | 2120-2140 | 2131 | 4 |
|  | GRMZM2G427444_T01 | 0.5 | 2119-2139 | 2130 | 4 |
|  | GRMZM2G427444_T04 | 0.5 | 13643-13663 | 13654 | 4 |
|  | GRMZM5G806488_T01 | 0.5 | 36778-36798 | 36789 | 4 |
| Predict-miR-113 | GRMZM2G100246_T01 | 2 | 1643-1663 | 1654 | 4 |
|  | GRMZM2G448151_T01 | 3.5 | 932-951 | 942 | 4 |
|  | GRMZM2G181002_T01 | 4 | 1416-1436 | 1427 | 4 |
| Predict-miR-137 | GRMZM2G100246_T01 | 2 | 1643-1663 | 1654 | 4 |
| Predict-miR-140 | GRMZM2G027756_T02 | 4 | 630-651 | 642 | 4 |
|  | GRMZM2G027756_T01 | 4 | 882-903 | 894 | 4 |
| Predict-miR-157 | GRMZM5G806488_T01 | 4 | 26496-26516 | 26508 | 4 |
|  | GRMZM2G072419_T01 | 4 | 675-695 | 687 | 4 |
|  | GRMZM2G161965_T01 | 4 | 736-756 | 748 | 4 |
|  | GRMZM2G176216_T01 | 4 | 7081-7101 | 7093 | 4 |
|  | GRMZM2G137648_T01 | 4 | 3606-3626 | 3618 | 4 |
| Predict-miR-36 | GRMZM2G455413_T01 | 2.5 | 218-237 | 228 | 2 |
|  | GRMZM2G427369_T01 | 2.5 | 251-270 | 261 | 2 |
|  | GRMZM5G844143_T01 | 2.5 | 215-234 | 225 | 2 |
|  | GRMZM2G427369_T02 | 2.5 | 89-108 | 99 | 2 |
| Predict-miR-4 | GRMZM2G004880_T01 | 3.5 | 1888-1909 | 1900 | 4 |
| Predict-miR-44 | GRMZM2G100246_T01 | 3.5 | 1643-1663 | 1654 | 4 |
| Predict-miR-56 | GRMZM5G813608_T01 | 4 | 1580-1600 | 1591 | 2 |
|  | GRMZM2G458099_T01 | 4 | 460-480 | 471 | 2 |
|  | GRMZM2G058612_T01 | 4 | 1001-1022 | 1013 | 4 |
| zma-miR156j-5p | GRMZM2G307588_T01 | 0 | 1000-1020 | 1011 | 4 |
|  | GRMZM2G101511_T02 | 0 | 1095-1115 | 1106 | 4 |
|  | AC233751.1_FGT002 | 0 | 1414-1434 | 1425 | 4 |
|  | GRMZM2G101511_T01 | 0 | 1201-1221 | 1212 | 4 |
|  | GRMZM2G052921_T01 | 1 | 276-296 | 287 | 4 |
|  | GRMZM2G163813_T04 | 1 | 1005-1025 | 1016 | 4 |
|  | GRMZM2G163813_T02 | 1 | 1233-1253 | 1244 | 4 |
|  | GRMZM2G113779_T01 | 2 | 318-338 | 329 | 4 |
|  | GRMZM2G156621_T01 | 2 | 858-878 | 869 | 4 |
|  | GRMZM2G126827_T01 | 2 | 926-946 | 937 | 4 |
| zma-miR156k-5p | GRMZM2G052921_T01 | 1 | 277-296 | 287 | 4 |
|  | GRMZM2G163813_T04 | 1 | 1006-1025 | 1016 | 4 |
|  | GRMZM2G307588_T01 | 1 | 1001-1020 | 1011 | 4 |
|  | GRMZM2G101511_T02 | 1 | 1096-1115 | 1106 | 4 |
|  | AC233751.1_FGT002 | 1 | 1415-1434 | 1425 | 4 |
|  | GRMZM2G163813_T02 | 1 | 1234-1253 | 1244 | 4 |
|  | GRMZM2G101511_T01 | 1 | 1202-1221 | 1212 | 4 |
|  | GRMZM2G113779_T01 | 2 | 319-338 | 329 | 4 |
|  | GRMZM2G156621_T01 | 2 | 859-878 | 869 | 4 |
|  | GRMZM2G126827_T01 | 2 | 927-946 | 937 | 4 |
| zma-miR156l-5p | GRMZM2G052921_T01 | 1 | 277-296 | 287 | 4 |
|  | GRMZM2G163813_T04 | 1 | 1006-1025 | 1016 | 4 |
|  | GRMZM2G307588_T01 | 1 | 1001-1020 | 1011 | 4 |
|  | GRMZM2G101511_T02 | 1 | 1096-1115 | 1106 | 4 |
|  | AC233751.1_FGT002 | 1 | 1415-1434 | 1425 | 4 |
|  | GRMZM2G163813_T02 | 1 | 1234-1253 | 1244 | 4 |
|  | GRMZM2G101511_T01 | 1 | 1202-1221 | 1212 | 4 |
|  | GRMZM2G113779_T01 | 2 | 319-338 | 329 | 4 |
|  | GRMZM2G156621_T01 | 2 | 859-878 | 869 | 4 |
|  | GRMZM2G126827_T01 | 2 | 927-946 | 937 | 4 |
| zma-miR159d-3p | GRMZM2G416652_T01 | 3 | 1200-1220 | 1211 | 0 |
|  | GRMZM2G416652_T02 | 3 | 1200-1220 | 1211 | 0 |
|  | GRMZM2G139688_T01 | 3 | 1893-1913 | 1904 | 0 |
|  | GRMZM2G167088_T01 | 3 | 1410-1430 | 1421 | 0 |
|  | AC209015.3_FGT004 | 4 | 328-348 | 339 | 0 |
|  | GRMZM2G004090_T01 | 4 | 1001-1021 | 1012 | 0 |
|  | GRMZM2G028054_T03 | 4 | 1693-1713 | 1704 | 0 |
|  | GRMZM2G028054_T02 | 4 | 1693-1713 | 1704 | 0 |
|  | GRMZM2G028054_T01 | 4 | 1693-1713 | 1704 | 0 |
|  | GRMZM2G113073_T01 | 4 | 121-141 | 132 | 2 |
| zma-miR159e-3p | GRMZM2G416652_T01 | 4 | 1200-1220 | 1211 | 0 |
|  | GRMZM2G416652_T02 | 4 | 1200-1220 | 1211 | 0 |
|  | GRMZM2G167088_T01 | 4 | 1410-1430 | 1421 | 0 |
| zma-miR159g-3p | GRMZM2G139688_T01 | 2 | 1893-1913 | 1904 | 0 |
|  | GRMZM2G416652_T01 | 4 | 1200-1220 | 1211 | 0 |
|  | GRMZM2G004090_T01 | 4 | 1001-1021 | 1012 | 0 |
|  | GRMZM2G416652_T02 | 4 | 1200-1220 | 1211 | 0 |
|  | GRMZM2G167088_T01 | 4 | 1410-1430 | 1421 | 0 |
| zma-miR159i-3p | GRMZM2G139688_T01 | 1.5 | 1893-1913 | 1904 | 0 |
|  | GRMZM2G416652_T01 | 3.5 | 1200-1220 | 1211 | 0 |
|  | GRMZM2G004090_T01 | 3.5 | 1001-1021 | 1012 | 0 |
|  | GRMZM2G416652_T02 | 3.5 | 1200-1220 | 1211 | 0 |
|  | GRMZM2G167088_T01 | 3.5 | 1410-1430 | 1421 | 0 |
|  | GRMZM2G423833_T01 | 4 | 905-925 | 916 | 0 |
|  | GRMZM2G028054_T03 | 4 | 1693-1713 | 1704 | 0 |
|  | GRMZM2G028054_T02 | 4 | 1693-1713 | 1704 | 0 |
|  | GRMZM2G028054_T01 | 4 | 1693-1713 | 1704 | 0 |
| zma-miR159k-3p | GRMZM2G416652_T01 | 1.5 | 1200-1220 | 1211 | 0 |
|  | GRMZM2G416652_T02 | 1.5 | 1200-1220 | 1211 | 0 |
|  | GRMZM2G167088_T01 | 1.5 | 1410-1430 | 1421 | 0 |
|  | GRMZM2G423833_T01 | 2 | 905-925 | 916 | 0 |
|  | AC209015.3_FGT004 | 2.5 | 328-348 | 339 | 0 |
|  | GRMZM2G004090_T01 | 2.5 | 1001-1021 | 1012 | 0 |
|  | GRMZM2G093789_T01 | 2.5 | 11002-11022 | 11013 | 2 |
|  | GRMZM2G028054_T03 | 3 | 1693-1713 | 1704 | 0 |
|  | GRMZM2G028054_T02 | 3 | 1693-1713 | 1704 | 0 |
|  | GRMZM2G028054_T01 | 3 | 1693-1713 | 1704 | 0 |
|  | GRMZM2G139688_T01 | 3.5 | 1893-1913 | 1904 | 0 |
|  | GRMZM5G897958_T01 | 4 | 1979-2000 | 1990 | 4 |
| zma-miR160f-3p | GRMZM5G875238_T01 | 4 | 375-395 | 386 | 2 |
|  | AC207656.3_FGT002 | 1 | 1350-1370 | 1361 | 0 |
|  | GRMZM2G390641_T02 | 1 | 1672-1692 | 1683 | 0 |
|  | GRMZM2G390641_T01 | 1 | 1705-1725 | 1716 | 0 |
|  | GRMZM2G153233_T01 | 1 | 1695-1715 | 1706 | 0 |
|  | GRMZM2G159399_T01 | 1 | 1785-1805 | 1796 | 0 |
|  | GRMZM2G081406_T01 | 2 | 1499-1519 | 1510 | 4 |
| zma-miR160g-5p | AC207656.3_FGT002 | 1 | 1350-1370 | 1361 | 0 |
|  | GRMZM2G390641_T02 | 1 | 1672-1692 | 1683 | 0 |
|  | GRMZM2G390641_T01 | 1 | 1705-1725 | 1716 | 0 |
|  | GRMZM2G153233_T01 | 1 | 1695-1715 | 1706 | 0 |
|  | GRMZM2G159399_T01 | 1 | 1785-1805 | 1796 | 0 |
|  | GRMZM2G081406_T01 | 2 | 1499-1519 | 1510 | 4 |
| zma-miR162-3p | GRMZM2G040762_T01 | 2 | 1461-1481 | 1471 | 4 |
| zma-miR164f-5p | GRMZM2G063522_T01 | 1 | 895-915 | 906 | 0 |
|  | GRMZM2G114850_T01 | 1 | 1180-1200 | 1191 | 4 |
|  | GRMZM2G063522_T01 | 2 | 895-915 | 906 | 0 |
|  | GRMZM2G114850_T01 | 2 | 1180-1200 | 1191 | 4 |
|  | GRMZM2G063522_T01 | 2.5 | 895-915 | 906 | 0 |
|  | GRMZM2G114850_T01 | 2.5 | 1180-1200 | 1191 | 4 |
| zma-miR166a-3p | GRMZM2G042250_T04 | 3 | 1190-1210 | 1201 | 0 |
|  | GRMZM2G109987_T04 | 3 | 1292-1312 | 1303 | 0 |
|  | GRMZM2G109987_T05 | 3 | 774-794 | 785 | 0 |
|  | GRMZM2G042250_T02 | 3 | 901-921 | 912 | 0 |
|  | GRMZM2G109987_T03 | 3 | 1292-1312 | 1303 | 0 |
|  | GRMZM2G042250_T01 | 3 | 1190-1210 | 1201 | 0 |
|  | GRMZM2G042250_T03 | 3 | 1190-1210 | 1201 | 0 |
|  | GRMZM2G109987_T01 | 3 | 1292-1312 | 1303 | 0 |
|  | GRMZM2G109987_T02 | 3 | 1292-1312 | 1303 | 0 |
|  | GRMZM2G038198_T01 | 4 | 485-505 | 496 | 0 |
| zma-miR166d-3p | GRMZM2G042250_T04 | 2 | 1191-1210 | 1201 | 0 |
|  | GRMZM2G109987_T04 | 2 | 1293-1312 | 1303 | 0 |
|  | GRMZM2G109987_T05 | 2 | 775-794 | 785 | 0 |
|  | GRMZM2G042250_T02 | 2 | 902-921 | 912 | 0 |
|  | GRMZM2G109987_T03 | 2 | 1293-1312 | 1303 | 0 |
|  | GRMZM2G042250_T01 | 2 | 1191-1210 | 1201 | 0 |
|  | GRMZM2G042250_T03 | 2 | 1191-1210 | 1201 | 0 |
|  | GRMZM2G109987_T01 | 2 | 1293-1312 | 1303 | 0 |
|  | GRMZM2G109987_T02 | 2 | 1293-1312 | 1303 | 0 |
|  | GRMZM2G038198_T01 | 3 | 486-505 | 496 | 0 |
| zma-miR166m-3p | GRMZM2G042250_T04 | 3 | 1190-1210 | 1201 | 0 |
|  | GRMZM2G109987_T04 | 3 | 1292-1312 | 1303 | 0 |
|  | GRMZM2G109987_T05 | 3 | 774-794 | 785 | 0 |
|  | GRMZM2G042250_T02 | 3 | 901-921 | 912 | 0 |
|  | GRMZM2G109987_T03 | 3 | 1292-1312 | 1303 | 0 |
|  | GRMZM2G042250_T01 | 3 | 1190-1210 | 1201 | 0 |
|  | GRMZM2G042250_T03 | 3 | 1190-1210 | 1201 | 0 |
|  | GRMZM2G109987_T01 | 3 | 1292-1312 | 1303 | 0 |
|  | GRMZM2G109987_T02 | 3 | 1292-1312 | 1303 | 0 |
|  | GRMZM2G038198_T01 | 4 | 485-505 | 496 | 0 |
| zma-miR166n-3p | GRMZM2G042250_T04 | 2.5 | 1191-1210 | 1201 | 0 |
|  | GRMZM2G109987_T04 | 2.5 | 1293-1312 | 1303 | 0 |
|  | GRMZM2G109987_T05 | 2.5 | 775-794 | 785 | 0 |
|  | GRMZM2G042250_T02 | 2.5 | 902-921 | 912 | 0 |
|  | GRMZM2G109987_T03 | 2.5 | 1293-1312 | 1303 | 0 |
|  | GRMZM2G042250_T01 | 2.5 | 1191-1210 | 1201 | 0 |
|  | GRMZM2G042250_T03 | 2.5 | 1191-1210 | 1201 | 0 |
|  | GRMZM2G109987_T01 | 2.5 | 1293-1312 | 1303 | 0 |
|  | GRMZM2G109987_T02 | 2.5 | 1293-1312 | 1303 | 0 |
|  | GRMZM2G038198_T01 | 3.5 | 486-505 | 496 | 0 |
| zma-miR167c-5p | GRMZM2G078274_T02 | 4 | 187-208 | 198 | 2 |
|  | GRMZM2G068328_T07 | 4 | 788-808 | 799 | 4 |
|  | GRMZM2G078274_T03 | 4 | 2500-2521 | 2511 | 0 |
|  | GRMZM2G078274_T01 | 4 | 2500-2521 | 2511 | 0 |
|  | GRMZM2G475882_T01 | 4 | 2530-2551 | 2541 | 0 |
|  | GRMZM2G475882_T02 | 4 | 2648-2669 | 2659 | 0 |
| zma-miR167g-3p | GRMZM2G005887_T09 | 4 | 422-440 | 431 | 4 |
|  | GRMZM2G005887_T02 | 4 | 355-373 | 364 | 4 |
|  | GRMZM2G005887_T04 | 4 | 422-440 | 431 | 4 |
|  | GRMZM2G005887_T06 | 4 | 299-317 | 308 | 4 |
|  | GRMZM2G005887_T08 | 4 | 386-404 | 395 | 4 |
|  | GRMZM2G005887_T05 | 4 | 439-457 | 448 | 4 |
|  | GRMZM2G005887_T01 | 4 | 542-560 | 551 | 4 |
|  | GRMZM2G005887_T03 | 4 | 422-440 | 431 | 4 |
| zma-miR167i-3p | GRMZM2G110881_T01 | 4 | 798-815 | 806 | 4 |
|  | GRMZM2G110881_T03 | 4 | 838-855 | 846 | 4 |
|  | GRMZM2G009808_T01 | 4 | 2007-2024 | 2016 | 4 |
| zma-miR167j-5p | GRMZM2G068328_T07 | 4 | 788-808 | 799 | 4 |
|  | GRMZM2G174255_T01 | 4 | 37-58 | 48 | 4 |
|  | GRMZM2G351990_T01 | 4 | 309-330 | 320 | 4 |
| zma-miR169b-5p | GRMZM2G000686_T06 | 3.5 | 890-910 | 901 | 4 |
|  | GRMZM2G000686_T04 | 3.5 | 962-982 | 973 | 4 |
|  | GRMZM2G000686_T08 | 3.5 | 1543-1563 | 1554 | 4 |
|  | GRMZM2G000686_T10 | 3.5 | 1560-1580 | 1571 | 4 |
|  | GRMZM2G000686_T05 | 3.5 | 1765-1785 | 1776 | 4 |
|  | GRMZM2G000686_T02 | 3.5 | 1816-1836 | 1827 | 4 |
|  | GRMZM2G000686_T03 | 3.5 | 1837-1857 | 1848 | 4 |
|  | GRMZM2G000686_T01 | 3.5 | 2120-2140 | 2131 | 4 |
| zma-miR169h | GRMZM2G000686_T06 | 4 | 890-910 | 901 | 4 |
|  | GRMZM2G000686_T04 | 4 | 962-982 | 973 | 4 |
|  | GRMZM2G000686_T08 | 4 | 1543-1563 | 1554 | 4 |
|  | GRMZM2G000686_T10 | 4 | 1560-1580 | 1571 | 4 |
|  | GRMZM2G000686_T05 | 4 | 1765-1785 | 1776 | 4 |
|  | GRMZM2G000686_T02 | 4 | 1816-1836 | 1827 | 4 |
|  | GRMZM2G000686_T03 | 4 | 1837-1857 | 1848 | 4 |
|  | GRMZM2G000686_T01 | 4 | 2120-2140 | 2131 | 4 |
| zma-miR169j-5p | GRMZM2G000686_T06 | 3 | 890-910 | 901 | 4 |
|  | GRMZM2G000686_T04 | 3 | 962-982 | 973 | 4 |
|  | GRMZM2G000686_T08 | 3 | 1543-1563 | 1554 | 4 |
|  | GRMZM2G000686_T10 | 3 | 1560-1580 | 1571 | 4 |
|  | GRMZM2G000686_T05 | 3 | 1765-1785 | 1776 | 4 |
|  | GRMZM2G000686_T02 | 3 | 1816-1836 | 1827 | 4 |
|  | GRMZM2G000686_T03 | 3 | 1837-1857 | 1848 | 4 |
|  | GRMZM2G000686_T01 | 3 | 2120-2140 | 2131 | 4 |
| zma-miR169l-5p | GRMZM2G000686_T06 | 4 | 890-910 | 901 | 4 |
|  | GRMZM2G000686_T04 | 4 | 962-982 | 973 | 4 |
|  | GRMZM2G000686_T08 | 4 | 1543-1563 | 1554 | 4 |
|  | GRMZM2G000686_T10 | 4 | 1560-1580 | 1571 | 4 |
|  | GRMZM2G000686_T05 | 4 | 1765-1785 | 1776 | 4 |
|  | GRMZM2G000686_T02 | 4 | 1816-1836 | 1827 | 4 |
|  | GRMZM2G000686_T03 | 4 | 1837-1857 | 1848 | 4 |
|  | GRMZM2G000686_T01 | 4 | 2120-2140 | 2131 | 4 |
| zma-miR169o-3p | GRMZM2G124288_T02 | 2.5 | 2297-2315 | 2306 | 4 |
|  | GRMZM2G124288_T01 | 2.5 | 2391-2409 | 2400 | 4 |
| zma-miR169p-3p | GRMZM2G021549_T01 | 4 | 1148-1168 | 1159 | 4 |
|  | GRMZM2G000686_T06 | 2.5 | 890-910 | 901 | 4 |
|  | GRMZM2G000686_T04 | 2.5 | 962-982 | 973 | 4 |
|  | GRMZM2G000686_T08 | 2.5 | 1543-1563 | 1554 | 4 |
|  | GRMZM2G000686_T10 | 2.5 | 1560-1580 | 1571 | 4 |
|  | GRMZM2G000686_T05 | 2.5 | 1765-1785 | 1776 | 4 |
|  | GRMZM2G000686_T02 | 2.5 | 1816-1836 | 1827 | 4 |
|  | GRMZM2G000686_T03 | 2.5 | 1837-1857 | 1848 | 4 |
|  | GRMZM2G000686_T01 | 2.5 | 2120-2140 | 2131 | 4 |
|  | GRMZM2G000236_T01 | 3.5 | 1230-1249 | 1240 | 2 |
|  | GRMZM2G106303_T01 | 4 | 1116-1135 | 1126 | 2 |
| zma-miR169r-5p | GRMZM2G000686_T06 | 2.5 | 890-910 | 901 | 4 |
|  | GRMZM2G000686_T04 | 2.5 | 962-982 | 973 | 4 |
|  | GRMZM2G000686_T08 | 2.5 | 1543-1563 | 1554 | 4 |
|  | GRMZM2G000686_T10 | 2.5 | 1560-1580 | 1571 | 4 |
|  | GRMZM2G000686_T05 | 2.5 | 1765-1785 | 1776 | 4 |
|  | GRMZM2G000686_T02 | 2.5 | 1816-1836 | 1827 | 4 |
|  | GRMZM2G000686_T03 | 2.5 | 1837-1857 | 1848 | 4 |
|  | GRMZM2G000686_T01 | 2.5 | 2120-2140 | 2131 | 4 |
| zma-miR171c-3p | GRMZM2G317338_T01 | 3 | 252-272 | 263 | 0 |
|  | GRMZM2G418899_T01 | 3 | 45-65 | 56 | 0 |
|  | GRMZM5G825321_T01 | 3 | 507-527 | 518 | 0 |
|  | GRMZM2G418899_T02 | 3 | 45-65 | 56 | 0 |
|  | GRMZM2G110579_T01 | 3 | 1078-1098 | 1089 | 0 |
|  | GRMZM2G098800_T01 | 3 | 920-940 | 931 | 2 |
|  | GRMZM2G037792_T01 | 3 | 1322-1342 | 1333 | 0 |
|  | GRMZM2G098800_T02 | 3 | 1391-1411 | 1402 | 2 |
|  | GRMZM5G825321_T02 | 3 | 1460-1480 | 1471 | 0 |
| zma-miR171f-3p | GRMZM2G317338_T01 | 3 | 249-269 | 260 | 4 |
|  | GRMZM2G418899_T01 | 3 | 42-62 | 53 | 2 |
|  | GRMZM5G825321_T01 | 3 | 504-524 | 515 | 3 |
|  | GRMZM2G418899_T02 | 3 | 42-62 | 53 | 2 |
|  | GRMZM2G110579_T01 | 3 | 1075-1095 | 1086 | 4 |
|  | GRMZM2G098800_T01 | 3 | 917-937 | 928 | 4 |
|  | GRMZM2G037792_T01 | 3 | 1319-1339 | 1330 | 3 |
|  | GRMZM2G098800_T02 | 3 | 1388-1408 | 1399 | 4 |
|  | GRMZM5G825321_T02 | 3 | 1457-1477 | 1468 | 3 |
| zma-miR171i-3p | GRMZM2G317338_T01 | 1 | 252-272 | 263 | 0 |
|  | GRMZM2G418899_T01 | 1 | 45-65 | 56 | 0 |
|  | GRMZM5G825321_T01 | 1 | 507-527 | 518 | 0 |
|  | GRMZM2G418899_T02 | 1 | 45-65 | 56 | 0 |
|  | GRMZM2G110579_T01 | 1 | 1078-1098 | 1089 | 0 |
|  | GRMZM2G098800_T01 | 1 | 920-940 | 931 | 2 |
|  | GRMZM2G037792_T01 | 1 | 1322-1342 | 1333 | 0 |
|  | GRMZM2G098800_T02 | 1 | 1391-1411 | 1402 | 2 |
|  | GRMZM5G825321_T02 | 1 | 1460-1480 | 1471 | 0 |
| zma-miR171m-3p | GRMZM2G317338_T01 | 1.5 | 252-272 | 263 | 0 |
|  | GRMZM2G418899_T01 | 1.5 | 45-65 | 56 | 0 |
|  | GRMZM5G825321_T01 | 1.5 | 507-527 | 518 | 0 |
|  | GRMZM2G418899_T02 | 1.5 | 45-65 | 56 | 0 |
|  | GRMZM2G110579_T01 | 1.5 | 1078-1098 | 1089 | 0 |
|  | GRMZM2G098800_T01 | 1.5 | 920-940 | 931 | 2 |
|  | GRMZM2G037792_T01 | 1.5 | 1322-1342 | 1333 | 0 |
|  | GRMZM2G098800_T02 | 1.5 | 1391-1411 | 1402 | 2 |
|  | GRMZM5G825321_T02 | 1.5 | 1460-1480 | 1471 | 0 |
| zma-miR171n-3p | GRMZM2G317338_T01 | 0 | 252-272 | 263 | 0 |
|  | GRMZM2G418899_T01 | 0 | 45-65 | 56 | 0 |
|  | GRMZM5G825321_T01 | 0 | 507-527 | 518 | 0 |
|  | GRMZM2G418899_T02 | 0 | 45-65 | 56 | 0 |
|  | GRMZM2G110579_T01 | 0 | 1078-1098 | 1089 | 0 |
|  | GRMZM2G098800_T01 | 0 | 920-940 | 931 | 2 |
|  | GRMZM2G037792_T01 | 0 | 1322-1342 | 1333 | 0 |
|  | GRMZM2G098800_T02 | 0 | 1391-1411 | 1402 | 2 |
|  | GRMZM5G825321_T02 | 0 | 1460-1480 | 1471 | 0 |
| zma-miR172c-3p | GRMZM2G416725_T01 | 1 | 400-419 | 410 | 0 |
|  | GRMZM5G862109_T01 | 1 | 1559-1578 | 1569 | 4 |
|  | GRMZM5G862109_T02 | 1 | 1685-1704 | 1695 | 4 |
|  | GRMZM2G076602_T01 | 3 | 926-945 | 936 | 0 |
|  | GRMZM2G174784_T01 | 3 | 2242-2261 | 2252 | 1 |
|  | GRMZM2G074436_T01 | 4 | 1001-1020 | 1011 | 2 |
|  | GRMZM5G879527_T04 | 4 | 1083-1103 | 1093 | 4 |
|  | GRMZM5G879527_T02 | 4 | 1933-1953 | 1943 | 4 |
|  | GRMZM5G879527_T03 | 4 | 2305-2325 | 2315 | 4 |
| zma-miR172e | GRMZM2G416725_T01 | 2.5 | 399-419 | 410 | 0 |
|  | GRMZM5G862109_T01 | 2.5 | 1558-1578 | 1569 | 4 |
|  | GRMZM5G862109_T02 | 2.5 | 1684-1704 | 1695 | 4 |
|  | GRMZM2G076602_T01 | 3 | 925-945 | 936 | 0 |
|  | GRMZM2G174784_T01 | 3 | 2241-2261 | 2252 | 1 |
| zma-miR319c-5p | GRMZM2G107595_T01 | 4 | 375-394 | 385 | 2 |
|  | GRMZM2G106108_T01 | 4 | 408-427 | 418 | 2 |
|  | GRMZM2G127426_T01 | 4 | 651-670 | 661 | 2 |
|  | GRMZM2G135978_T01 | 1 | 1721-1741 | 1732 | 4 |
|  | GRMZM5G848945_T02 | 1 | 1737-1757 | 1748 | 2 |
|  | GRMZM2G137451_T01 | 3.5 | 1678-1699 | 1690 | 2 |
|  | GRMZM2G137451_T02 | 3.5 | 1973-1994 | 1985 | 2 |
| zma-miR394b-5p | GRMZM2G119650_T01 | 0 | 1227-1246 | 1237 | 2 |
|  | GRMZM2G064954_T01 | 0 | 1405-1424 | 1415 | 0 |
| zma-miR395k-3p | GRMZM2G149952_T01 | 4 | 453-473 | 464 | 2 |
| zma-miR395m-3p | GRMZM2G149952_T01 | 0.5 | 453-473 | 464 | 2 |
|  | GRMZM2G051270_T02 | 1.5 | 398-418 | 409 | 2 |
|  | GRMZM2G051270_T03 | 1.5 | 528-548 | 539 | 2 |
|  | GRMZM2G051270_T01 | 1.5 | 616-636 | 627 | 2 |
| zma-miR395o-3p | GRMZM2G149952_T01 | 2 | 453-473 | 464 | 2 |
|  | GRMZM2G051270_T02 | 3 | 398-418 | 409 | 2 |
|  | GRMZM2G051270_T03 | 3 | 528-548 | 539 | 2 |
|  | GRMZM2G051270_T01 | 3 | 616-636 | 627 | 2 |
| zma-miR395p-3p | GRMZM2G149952_T01 | 1 | 453-473 | 464 | 2 |
|  | GRMZM2G051270_T02 | 2 | 398-418 | 409 | 2 |
|  | GRMZM2G051270_T03 | 2 | 528-548 | 539 | 2 |
|  | GRMZM2G051270_T01 | 2 | 616-636 | 627 | 2 |
| zma-miR396a-5p | GRMZM2G067743_T02 | 3 | 538-559 | 549 | 1 |
|  | GRMZM2G067743_T03 | 3 | 537-558 | 548 | 1 |
|  | GRMZM2G067743_T01 | 3 | 661-682 | 672 | 1 |
|  | GRMZM2G099862_T02 | 3 | 557-578 | 568 | 0 |
|  | GRMZM2G018414_T02 | 3 | 480-501 | 491 | 0 |
|  | GRMZM2G099862_T03 | 3 | 643-664 | 654 | 0 |
|  | GRMZM2G129147_T01 | 3 | 639-660 | 650 | 4 |
|  | GRMZM2G099862_T01 | 3 | 690-711 | 701 | 0 |
|  | GRMZM2G018414_T01 | 3 | 742-763 | 753 | 0 |
|  | GRMZM2G129147_T02 | 3 | 819-840 | 830 | 4 |
|  | GRMZM2G119359_T01 | 3 | 811-832 | 822 | 1 |
|  | GRMZM2G099862_T04 | 3 | 444-465 | 455 | 0 |
|  | GRMZM5G853392_T04 | 3.5 | 9988-10009 | 9999 | 4 |
|  | GRMZM2G178261_T04 | 3.5 | 570-591 | 581 | 4 |
|  | GRMZM5G853392_T03 | 3.5 | 382-403 | 393 | 4 |
|  | GRMZM2G178261_T05 | 3.5 | 204-225 | 215 | 4 |
|  | GRMZM2G178261_T03 | 3.5 | 542-563 | 553 | 4 |
|  | GRMZM2G178261_T02 | 3.5 | 542-563 | 553 | 4 |
|  | GRMZM5G853392_T02 | 3.5 | 1120-1141 | 1131 | 4 |
|  | GRMZM2G443903_T01 | 3.5 | 2337-2358 | 2348 | 4 |
|  | GRMZM2G178261_T01 | 3.5 | 999-1020 | 1010 | 4 |
| zma-miR396e-3p | GRMZM2G031660_T01 | 4 | 990-1009 | 1000 | 4 |
| zma-miR396f-5p | GRMZM2G012631_T01 | 4 | 1568-1588 | 1579 | 2 |
|  | GRMZM5G853392_T04 | 4 | 9988-10009 | 9999 | 4 |
|  | GRMZM2G067743_T02 | 4 | 538-559 | 549 | 1 |
|  | GRMZM2G178261_T04 | 4 | 570-591 | 581 | 4 |
|  | GRMZM2G099862_T02 | 4 | 557-578 | 568 | 0 |
|  | GRMZM2G018414_T02 | 4 | 480-501 | 491 | 0 |
|  | GRMZM2G099862_T03 | 4 | 643-664 | 654 | 0 |
|  | GRMZM2G129147_T01 | 4 | 639-660 | 650 | 4 |
|  | GRMZM2G067743_T03 | 4 | 537-558 | 548 | 1 |
|  | GRMZM5G853392_T03 | 4 | 382-403 | 393 | 4 |
|  | GRMZM2G178261_T05 | 4 | 204-225 | 215 | 4 |
|  | GRMZM2G067743_T01 | 4 | 661-682 | 672 | 1 |
|  | GRMZM2G099862_T01 | 4 | 690-711 | 701 | 0 |
|  | GRMZM2G018414_T01 | 4 | 742-763 | 753 | 0 |
|  | GRMZM2G129147_T02 | 4 | 819-840 | 830 | 4 |
|  | GRMZM2G443903_T01 | 4 | 2337-2358 | 2348 | 4 |
|  | GRMZM2G119359_T01 | 4 | 811-832 | 822 | 1 |
|  | GRMZM2G178261_T03 | 4 | 542-563 | 553 | 4 |
|  | GRMZM2G178261_T02 | 4 | 542-563 | 553 | 4 |
|  | GRMZM2G099862_T04 | 4 | 444-465 | 455 | 0 |
|  | GRMZM5G853392_T02 | 4 | 1120-1141 | 1131 | 4 |
|  | GRMZM2G178261_T01 | 4 | 999-1020 | 1010 | 4 |
| zma-miR396h | GRMZM2G096169_T02 | 3.5 | 998-1019 | 1010 | 4 |
|  | GRMZM2G096169_T01 | 3.5 | 1122-1143 | 1134 | 4 |
| zma-miR398b-3p | GRMZM2G103812_T02 | 3 | 374-395 | 386 | 4 |
|  | GRMZM2G023847_T01 | 3 | 850-870 | 861 | 2 |
| zma-miR444b | GRMZM2G492156_T01 | 0 | 96-116 | 107 | 0 |
|  | GRMZM2G001024_T03 | 3.5 | 1374-1394 | 1385 | 0 |
|  | GRMZM2G001024_T02 | 3.5 | 1360-1380 | 1371 | 0 |
|  | GRMZM2G001024_T01 | 3.5 | 1378-1398 | 1389 | 0 |
| zma-miR482-3p | GRMZM2G064845_T01 | 4 | 1194-1213 | 1204 | 2 |
| zma-miR528b-5p | GRMZM2G107562_T01 | 2.5 | 570-589 | 580 | 0 |
| zma-miR529-3p | GRMZM2G153924_T01 | 4 | 3232-3252 | 3243 | 4 |
|  | GRMZM2G153924_T02 | 4 | 3391-3411 | 3402 | 4 |

Supplemental Table. 4 Primers used in the miRNAs and target genes validation.

| Primer | Primer sequence(5'-3') |
| --- | --- |
| miR171 | 5' TGACTGAGCCGTGCCAATATC 3' |
| miR169 | 5' CAGCCAAGGATGACTTGCCGA 3' |
| miR396 | 5' TTCCACAGCTTTCTTGAACTT 3' |
| miR166 | 5' TCGGACCAGGCTTCATTCCCC 3' |
| miR159 | 5' CTTGGATTGAAGGGAGCTCCT 3' |
| miR394 | 5' TTGGCATTCTGTCCACCTCC 3' |
| miR529 | 5' GCTGTACCCTCTCTCTTCTTC 3' |
| miR167 | 5' TGAAGCTGCCAGCATGATCTG 3' |
| 18S-A | 5' CCTGCGGCTTAATTGACTC 3' |
| 18S-S | 5' GTTAGCAGGCTGAGGTCTCG 3' |
| TmiR169（GRMZM2G000686）S | S: 5' CAAGAAGCGTCTGGAATGA3' |
| TmiR169（GRMZM2G000686）A | A: 5' GTATGATATGGCGAAGGACTG 3 |
| TmiR396（GRMZM2G012631）S | S: 5' GCTGCTCACTTCTGGCTTTA 3' |
| TmiR396（GRMZM2G012631）A | A: 5' CGACCTCCTCCATCTTGCT 3' |
| TmiR171（GRMZM2G098800）S | S: 5' ATTATGTGGGATTAGGTAGATGAGG3' |
| TmiR171（GRMZM2G098800）A | A: 5' ACAGCGTCTTCCCGTCTCC3' |
| TmiR166（GRMZM2G109987）S | S: 5' TCTGCTTCCCACCACCCAC 3' |
| TmiR166（GRMZM2G109987）A | A: 5' CCTTATCAAATCCTCCGCTAT 3' |
| TmiR159（GRMZM2G004090）S | S: 5' AGCAACCGCAACAAGACA 3' |
| TmiR159（GRMZM2G004090）A | A: 5' GTAGGCACCGACTCACTCC 3' |
| TmiR394（GRMZM2G119650）S | S: 5' TTGTAGGGATGGTGTTGAATG 3' |
| TmiR394（GRMZM2G119650）A | A: 5' GAAGTTTGATGCTTGGAGGA 3' |
| TmiR529（GRMZM2G153924）S | S: 5' GAAGCAGTCCCATCTCGTC 3' |
| TmiR529（GRMZM2G153924）A | A: 5' CAACTCCGTGGCACCGTAA 3' |
| TmiR167（GRMZM2G322348）S | S: 5' CCTGCTCATTTCTCGGTTAT 3' |
| TmiR167（GRMZM2G322348）A | A: 5' CGTCAATCTTCAGTCGGGTT 3' |

Note: The primer directions of the selected miRNA target genes were labeled with A and S, which mains Antisense (or Reverse) and Sense (or Forward), respectively. T means target.
