# Supplementary figures and images for: microRNA-dependent gene regulatory networks in maize leaf senescence
Source: BMC Plant Biol. 2016 Mar 22;16:73. doi: 10.1186/s12870-016-0755-y (PMC4802599; doi:10.1186/s12870-016-0755-y)

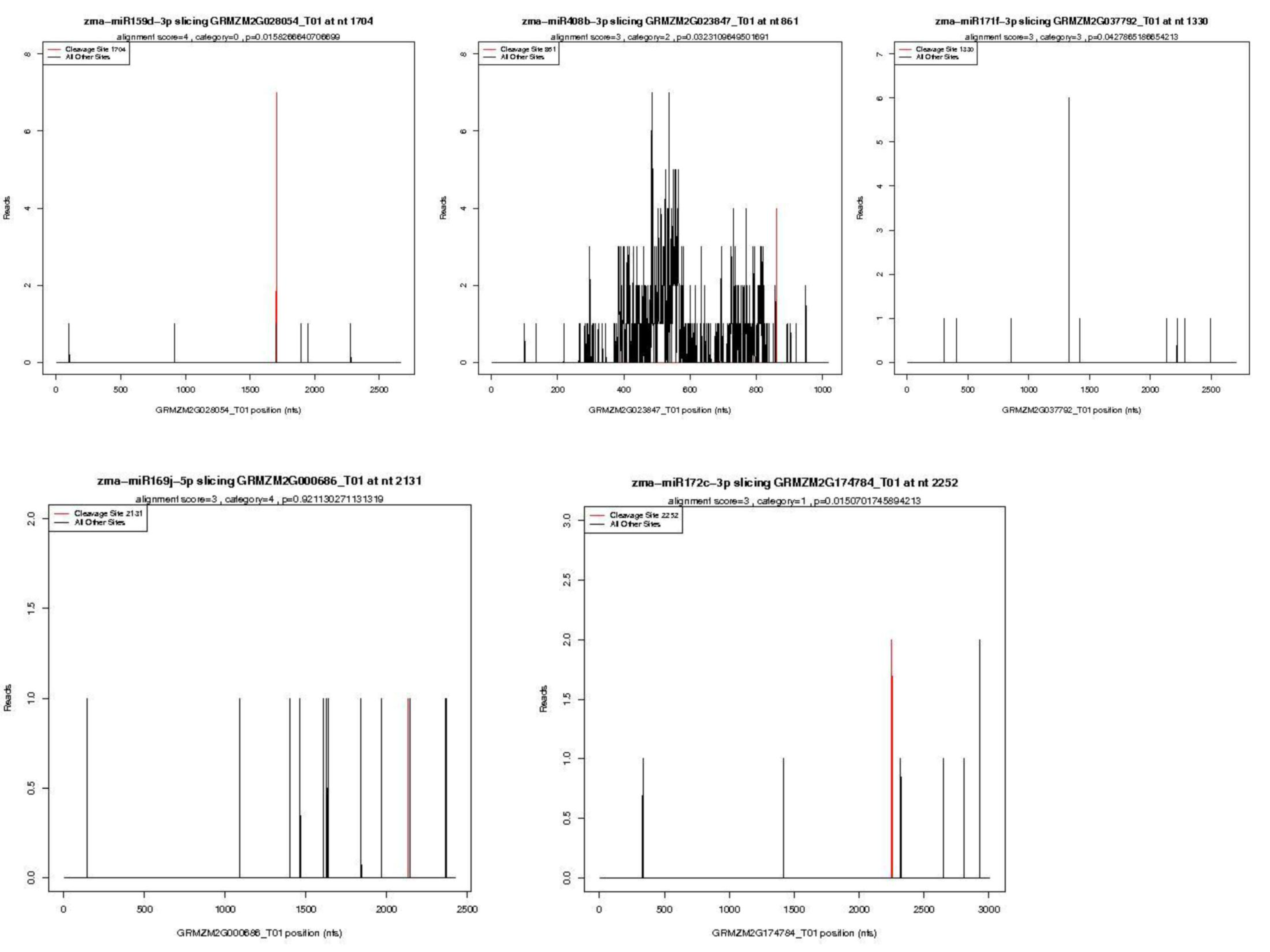

Supplement: Additional file 2: Figure S1. — T-plot of miRNA target genes. Five different categories of T-plots are shown. The degradome tag distributions along the target mRNA sequence are exhibited. The red line represents the sliced target transcripts. (TIF 4679 kb) [file 12870_2016_755_MOESM2_ESM.tif]
